# The telomere-to-telomere genome and comparative analysis from algae to higher plants reveal origin and evolution of biosynthesis of chlorogenic acid pathway and production in ramie (*Boehmeria nivea* L.)

**DOI:** 10.1093/hr/uhag144

**Published:** 2026-04-16

**Authors:** Song Gao, Qian Lin, Yangyu Tao, Jie Guo, Yuwei Guo, Hangfan Niu, Xintong Zhou, Yifan Ding, Fu Li, Touming Liu, Yanzhou Wang, Jianrong Chen

**Affiliations:** Key Laboratory of Biobreeding for Specialty Horticultural Crops of Jiangsu Province, College of Horticulture and Landscape Architecture, Yangzhou University, No. 88, Southern road of Daxue, 225009, Yangzhou, China; Hunan Provincial Key Laboratory of the Traditional Chinese Medicine Agricultural Biogenomics, Changsha Medical University, No. 1501 Leifeng Avenue, 410219, Changsha, Hunan, China; Institute of Bast Fiber Crops, Chinese Academy of Agricultural Sciences, No. 348, Western road of Xiajiahu, 410205, Changsha, China; Key Laboratory of Biobreeding for Specialty Horticultural Crops of Jiangsu Province, College of Horticulture and Landscape Architecture, Yangzhou University, No. 88, Southern road of Daxue, 225009, Yangzhou, China; Key Laboratory of Biobreeding for Specialty Horticultural Crops of Jiangsu Province, College of Horticulture and Landscape Architecture, Yangzhou University, No. 88, Southern road of Daxue, 225009, Yangzhou, China; Key Laboratory of Biobreeding for Specialty Horticultural Crops of Jiangsu Province, College of Horticulture and Landscape Architecture, Yangzhou University, No. 88, Southern road of Daxue, 225009, Yangzhou, China; Key Laboratory of Biobreeding for Specialty Horticultural Crops of Jiangsu Province, College of Horticulture and Landscape Architecture, Yangzhou University, No. 88, Southern road of Daxue, 225009, Yangzhou, China; Key Laboratory of Biobreeding for Specialty Horticultural Crops of Jiangsu Province, College of Horticulture and Landscape Architecture, Yangzhou University, No. 88, Southern road of Daxue, 225009, Yangzhou, China; Key Laboratory of Biobreeding for Specialty Horticultural Crops of Jiangsu Province, College of Horticulture and Landscape Architecture, Yangzhou University, No. 88, Southern road of Daxue, 225009, Yangzhou, China; Key Laboratory of Biobreeding for Specialty Horticultural Crops of Jiangsu Province, College of Horticulture and Landscape Architecture, Yangzhou University, No. 88, Southern road of Daxue, 225009, Yangzhou, China; Key Laboratory of Biobreeding for Specialty Horticultural Crops of Jiangsu Province, College of Horticulture and Landscape Architecture, Yangzhou University, No. 88, Southern road of Daxue, 225009, Yangzhou, China; Institute of Bast Fiber Crops, Chinese Academy of Agricultural Sciences, No. 348, Western road of Xiajiahu, 410205, Changsha, China; School of Biological and Chemical Engineering, Changsha University, No. 98 Hongshan Road, 410022, Changsha, China

## Abstract

Chlorogenic acid is an important secondary metabolite in plants, and its antioxidant and antimicrobial activities confers its remarkable medical value. Ramie (*Boehmeria nivea*), an important fiber crop, riches numerous Chlorogenic acid in the root and leaf, making it used as a traditional Chinese medicinal herb and livestock feed. In this study, we report a high-quality telomere-to-telomere (T2T) and gap-free genome of ramie. The assembly length was ~344 Mb, with all 28 telomeres and 14 centromeres. A total of 25 853 genes were identified, 90.5% of which were achieved functional annotation. Based on the T2T genome, we investigated the origin and evolution of the chlorogenic acid biosynthesis pathway and its production using comparative genomic and phylogenetic analyses. Subsequently, we identified five potential key genes involved in its biosynthesis via transcriptomic and metabolomic analyses, and characterized the metabolic pathway of chlorogenic acid biosynthesis in ramie through protease activity assays. Our T2T assembly represents the complete reference of genome and provides a valuable resource for genome and genetic studies in ramie; the proposed metabolic pathway of chlorogenic acid biosynthesis provided a basis for the improvement of chlorogenic acid content in ramie breeding.

## Introduction

As one of the important secondary metabolites and antioxidant substances in plants, chlorogenic acid is a kind of condensed phenolic acid generated by caffeic acid and quinic acid and belongs to phenylpropanoid class of compounds [1, 2]. Chlorogenic acid has various kinds of biological activities and has also been shown to have antioxidant activity, antimicrobial ability, cardiovascular protection, gene mutation inhibition, anti-tumor, anti-Human Immunodeficiency Virus, antihypertensive, antiviral, anti-inflammatory and other activities, as well as for in the treatment of gastric ulcers and diabetes mellitus and so on, it has also demonstrated a better and significant efficacy [3]; at the same time, it could scavenge free radicals and protect the cells from oxidative damage, which can help to resist aging and prevent a variety of diseases, and it has been applied in the fields of medicine, health care, food, and animal feeds, etc. [4].

Ramie (*Boehmeria nivea* L.) is a perennial herbaceous plant and belonging to the *Boehmeria* genus of the Urticaceae family. It is believed to have originated in China, where archeological evidence suggests it has been cultivated for over 4700 years [5]. It has been widely cultivated in China for its fibers, which are extracted from the stem bark, and it is a popular natural fiber in the textile industry [6]. Ramie is a fast-growing perennial plant cultivated in China as a source of bast fiber and nutritious green fodder suitable for all categories of farm animals [7]. Previous studies on cattle, sheep, pigs, horses, and poultry have highlighted the importance of ramie as a valuable nutritional resource in its green fodder form [8]. Simultaneously, ramie has been traditionally used for medicinal purposes and is recorded as a medicinal plant in the Compendium of Materia Medica because it contains many pharmacological components, such as chlorogenic acid (CGA) and total flavonoid substances [9]. The CGA content in ramie leaves is 439.49 μg/g, which gives them an impressive antioxidant capacity [10]. A recent study suggests that feeding laying hens food containing 3% ramie leaves significantly reduces malondialdehyde content and increases total antioxidant capacity in egg yolks [11]. Due to the high CGA content in ramie leaves, numerous CGA extraction strategies have been developed [12, 13], which are useful for fully exploiting ramie’s medicinal properties [14, 15]. For medicinal purposes, a higher CGA content in ramie is preferred. However, the limited understanding of CGA accumulation in ramie restricts improvements to its levels in this crop.

Despite the fact that four chromosome-level assemblies have been reported in ramie, these assemblies comprise at least 450 contigs, indicating relatively poor continuity [16, 17]. Recently, the development of HiFi high-fidelity long-read-length and Oxford Nanopore Technologies (ONT) ultra-long-read-length sequencing technologies has made it feasible to obtain high-quality genome assemblies with complete, contiguous, and accurate sequences. Numerous gapless, telomere-to-telomere (T2T) reference genomes have recently been assembled, providing new insights into the resolution of chromosomal regions known as ‘dark matter’ regions [18–20]. Previous studies indicated that the ‘1380’ cultivar possesses thin stem bark, low fiber content, and significantly higher pectin levels than other varieties. It also represents a potential high-protein feed source and is a superior forage ramie germplasm [16]. Here, we *de novo* assembled a gap-free, T2T ramie genome using HiFi and ONT sequencing, representing the first complete genome assembly of this species. Additionally, we examined the origins and evolution of the CGA biosynthetic pathway using multi-omics analysis.

## Results

### Genome assembly

**Figure 1:**
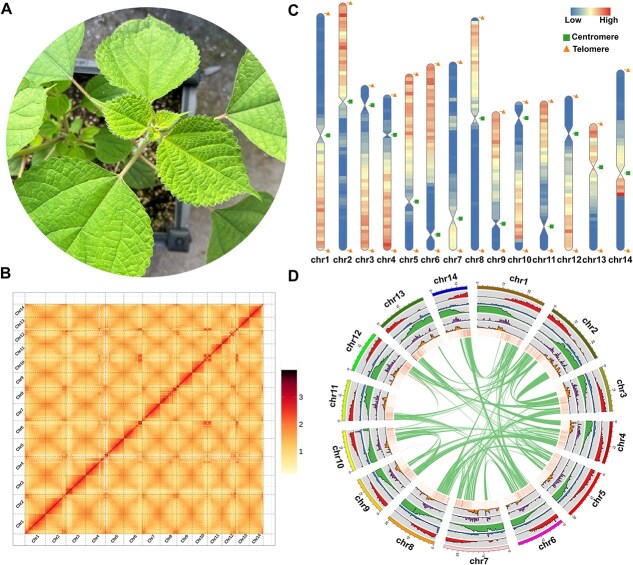
Genome assembly and genomic characterization of *Bniv_*1380. (A) Picture of *Bniv_*1380. (B) The HIC interaction matrix based on the assembly. (C) *Bniv_*1380 genome gene density and telomere and centromeres distribution. (D) The genomic features of *Bniv_*1380, in order from inside to outside, are genomic covariate site (genomic sequence homology block) distribution, Tandem Repeat density, Transposon Element coverage, Long Terminal Repeat Sequence (LTR) Gypsy coverage, LTR Copia coverage, Transposon Element coverage, GC density, and gene density.

We used the ramie germplasm ‘1380’ for the genome assembly (Fig. 1A). First, we performed Illumina sequencing and utilized the generated 86.08 million reads for K-mer survey analysis (k = 21). This analysis suggested that the genome size of the ‘1380’ germplasm is **~**350 Mb, with a heterozygosity rate of 2.05% and a repeat sequence length of ~180.72 Mb (Table S1, Fig. S1). Next, we performed PacBio and ONT sequencing, yielding 0.55 million HiFi reads and 53.23 million ONT reads. The total length of the HiFi and ONT reads was 8.96 and 55.09 Gb, respectively, with over 25.6× and 157× genome coverage (Table S2). We then assembled the HiFi reads using Hifiasm software to obtain a primary assembly containing 512 contigs with a cumulative size of 407 Mb. The contigs’ largest length and N50 length were 22.02 Mb and 7.12 Mb, respectively (Table 1).

**Table 1:** Statistics of genome assembly among *Bniv_*1380.

**Statistics without reference**	***Bniv_*1380 (HIFI-contig)**	***Bniv_*1380 (Gap-free)**	***Bniv*_QY**	***Bniv*_ZSZ1**
Number of contigs	512	14	450	657
Largest contig (Mb)	22.02	34.24		
Total length (Mb)	407.23	344.21	270.21	266.6
N50 (Mb)	7.12	24.40	10.51	2.33
L50	16	7		
N90 (Mb)	9.38	17.54		
L90	77	14		
Gap Number		0		

Note: *Bniv*_QY and *Bniv*_ZSZ1 are the reported assembly for wild Qingyezhuma and cultivated Zhongsizhu 1, respectively [16].

Next, we used Hi-C sequencing reads (30.77 Gb in length with 88× coverage) to assign contigs to chromosomes. The Hi-C mounting rate was 92.6%, producing in 14 pseudo-chromosomes (Fig. 1B). We then used ONT reads to fill the gaps in each chromosome with the tgsgapcloser software [21], resulting in a gap-free reference genome named *Bniv*_1380. The total length of the gap-free Bniv_1380 was 344 Mb, with N50 and N90 lengths of 24.40 and 17.54 Mb, respectively (Table 1, Fig. 1B). Finally, we searched the *Bniv*_1380 using the conserved telomeric repeat sequence (5’-CCCCTAAA/TTTAGGG-3′) and identified all 28 telomeres in the 14 chromosomes (Fig. 1C). This result implied that our novel assembly is a T2T version. Additionally, we identified all 14 centromeres using the quartTeT tool for centromere repeat identification [22]. Interestingly, the centromere positions indicated that all chromosomes except 1, 8, and 14 were acrocentric (Fig. 1C). Overall, our results suggest that the novel *Bniv*_1380 assembly is gap-free and T2T.

### Assessment of the genome quality

We used three methods to evaluate the quality of the *Bniv_*1380. First, we mapped the Illumina and HiFi reads, which were sequenced from the ‘1380’ germplasm, to the assembled genome. The results showed that 98.73% of the Illumina reads and 99.81% of the HiFi reads could be aligned to *Bniv*_1380. These reads covered 99.95% and 99.96%, respectively, of the genome region, indicating a high degree of genome sequence identity. Next, we estimated the genomic long-terminal repeat (LTR) assembly index (LAI) and found that the LAI of the novel assembly was 21.74, classifying *Bniv*_1380 as golden grade. Finally, we conducted a BUSCO assessment (viridiplantae_odb10) to demonstrate the integrity of the novel assembly. The results showed that 99.8% of BUSCO conserved genes were detected in Bniv_1380, including 419 complete, single-copy BUSCOs and five complete, duplicated BUSCOs (Table 2, Fig.  S2). Overall, our results suggested that *Bniv*_1380 is a high-quality assembly that could serve as a reference genome for future ramie genetic, genomic, and breeding studies.

**Table 2:** Quality assessment for genome assembly of *Bniv_*1380.

	***Bniv_*1380 (Gap-free)**	***Bniv*_QY**	***Bniv*_ZSZ1**	**Previous version by Liu *et al.* [** **23** **]**	**Previous version by Luan *et al.* [** **24** **]**
Mapping rate (%)	99.96	98.2	98.6		95.4
LTR assembly index	21.74	23.38	19.28		
Complete BUSCOs (%)	99.8	96.9	96.9		90.5
Complete and single-copy BUSCOs	98.6	93.9	94.2		84.2
Complete and duplicated copy BUSCOs	1.2%	3	2.7		6.3

Note: *Bniv*_QY and *Bniv*_ZSZ1 are wild and cultivated species, respectively [16].

### Genome annotation

Repetitive sequences, gene structures, functions, and non-coding RNAs were annotated. Our results showed that 227.93 Mb of repetitive sequences were identified in *Bniv*_1380, accounting for 61.19% of the ramie genome. Among them, miniature inverted repeat transposable element and LTR were the most abundant repetitive sequences, accounting for 4.68% and 16.81% of the genome, respectively (Table S3). We also combined homology prediction, *de novo* prediction, transcriptome-based prediction, and evidence integration to identified genes in *Bniv_*1380. This resulted in the identification of 25 853 putative genes. The mean coding sequence size was 3098.7 bp, and the average number of exons in gene and average exon length was 4.89 and 280.7 bp, respectively (Table S4). Compared the reported genome of wild Qingyezhuma (*Bniv*_QY) and cultivated Zhongsizhu 1 (*Bniv*_ZSZ1), there were over five thousand novel genes identified in our assembly, which caused that the distribution of length in coding sequence and exon exhibited slight difference (Fig. uew  S3). Moreover, ~90.52% of the putative genes were achieved functional annotation by searching against the six protein databases. In addition, 3325 non-coding RNAs were predicted in *Bniv_*1380 (Table S5).

### Evolution of ramie genome

**Figure 2:**
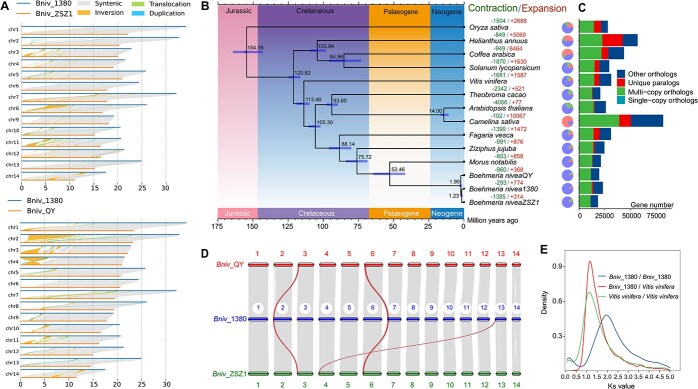
Phylogenetic and collinearity analysis of the ramie genome (*Bniv_*1380 T2T) with relatives’ plant species. (A) Structural variations between *Bniv*_ZSZ1 (*Bniv*_QY) and vT2T genomes. (B) Phylogenetic tree of 12 plant species. Time of divergence (MYA) is shown on the nodes. (C) Distribution of genes in all gene families of the 12 plant species. (D) Co-linearity of the *Bniv_*1380 and *Bniv*_ZSZ1 and *Bniv*_QY genomes. (E) WGD analysis diagram.

To exhibit an evolutionary comparison of ramie genome, we identified homologous genes of 14 species using the OrthoFinder [25]. A total of 36 767 gene families, including 456 602 genes were identified. Of these, 14 133 and 8236 orthologous families were species-specific and core, respectively (Table S6, Fig.  S4). Previous study suggested more contracted gene families compared to the expanded families in the genomes of wild and cultivated ramie [16]. Unlike the reported genomes of ramie, this study identified 774 expanded gene families in *Bniv_*1380, which is more than that of contracted gene families (Fig. 2A). The differential findings should result from more novel genes detected in *Bniv_*1380. Interestingly, numerous fiber growth genes displayed an expansion in ramie genome, such as *COMT1* (two genes), cellulose synthase *CSLD* (six genes), *IRX12* (four genes), *IRX15* (two genes), and MYB52 (three genes) (Table S7). Additionally, at least 113 expanded genes were potentially involved in the auxin signal pathway (Table S8). Functional enrichment of these expanded genes demonstrated that they significantly enriched in ‘Plant hormone signal transduction’ (*P* = 3.10 × 10^−20^; Fig.  S5). Their expansions of these auxin-related genes might be for meeting to the evolution of fast growth in ramie.

Compared the previous *Bniv_*ZSZ1 and *Bniv_*QY genomes with the *Bniv_*1380 genome, the collinear analysis diagram showed some structural variants of chromosomes in the ramie genomes of the three varieties (Fig. 2A). We also constructed a phylogenetic tree using the single-copy gene families and revealed that ramie showed the closest relationship with *Fragaria vesca*, *Ziziphus jujuba* and *Morus notabilis*, and the diverged time between them and ramie was ~8.81, 7.57, and 5.25 million years ago (MYA), respectively (Fig. 2B). Notably, the phylogenetic position of ‘1380’ germplasm was identified to fall between wild and cultivated ramie (Fig. 2B), suggesting it was a landrace without improvement.

Together with ramie, 11 species genomes (*Oryza sativa, Helianthus annuus, Coffea arabica, Solanum lycopersicum, Vitis vinifera, Theobroma cacao, Arabidopsis thaliana, Camelina sativa, Fagaria vesca, Ziziphus jujuba, and Morus notabilis*) were selected and analyzed for gene family clustering, unique paralogs, number of single-copy genes and multi-copy genes (Fig. 2C). We detected 1385 unique paralogs in *Bniv_*1380, these genes were distributed across every chromosome, but only 97 and 72 in reported *Bniv*_ZSZ1 and *Bniv*_QY genome, respectively, suggesting that our T2T genome gained new genes (Fig. 2C, Table S9, Fig.  S6). Notably, these unique genes are functionally enriched in ‘Phenylpropanoid biosynthesis,’ ‘Stilbenoid, diarylheptanoid and gingerol biosynthesis’, and ‘Flavonoid biosynthesis’ pathways, all belonging to secondary metabolism (Fig.  S6). There were distinct assembled errors in two telomere regions in previous versions (Fig. 2D). Based on the analysis of the synonymous mutation rate of homologous genes, we found that a separate genome-wide duplication event occurred in Ramie at a Ks value of ~2.0 (Fig. 2E).

### Evolution of chlorogenic acid pathway in plants

**Figure 3:**
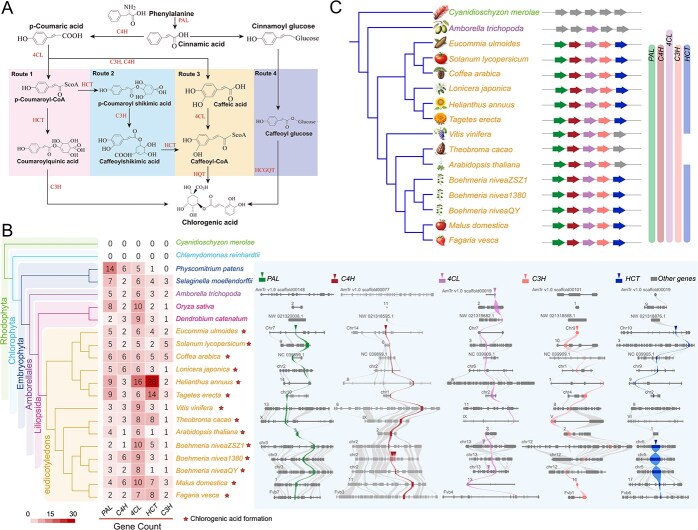
The discovery and evolution of a conserved chlorogenic acid synthase gene in plants. **(**A) A diagram showing the regulation of chlorogenic acid biosynthesis genes in the biosynthetic pathway. (B) A phylogenetic tree of 21 representative species from the four major branches of Rhodophyta, Chlorophyta, Embryophyta, Amborellales, Liliopsida, and eudicotyledons. A comprehensive comparative analysis of genomic regions revealed the evolutionary process of chlorogenic acid formation in plants. (C) An evolutionary model outlining the formation process of the chlorogenic acid biosynthesis pathway in plants. Green, red, purple, orange, and dark blue arrows represent *PAL, C4H, 4CL, C3H,* and *HCT*, respectively. Gray arrows represent the corresponding genes that are missing from the genome.

Numerous studies have reported on the potential biosynthetic pathways of chlorogenic acid involving four metabolic pathways (Fig. 3A) [26]. However, the evolutionary history of this pathway in plants remains unclear. Previous studies have shown that chlorogenic acid is present and abundant in various plants, including coffee, chrysanthemum, honeysuckle, eucommia, and sunflower [27]. In this study, we screened 21 representative plant species [28], including the aforementioned five, for homologous genes involved in the chlorogenic acid biosynthetic pathway (Fig. 3B). Our results revealed that all plant genomes contain genes involved in chlorogenic acid biosynthesis, except for the two algal plants *Cyanidioschyzon merolae* and *Chlamydomonas reinhardtii*. Subsequently, we performed collinearity analysis on key genes in the chlorogenic acid synthesis pathway. We observed that *PALs, C4Hs, 4CLs, C3Hs,* and *HCTs* were all located in collinearity blocks in species with high chlorogenic acid content, suggesting that they may have conserved biological functions, which ensures efficient chlorogenic acid biosynthesis (Fig. 3B).

Key genes in the chlorogenic acid synthesis pathway have been identified in Chlorophyta, Embryophyta, Amborellales, and Liliopsida plants. However, with the exception of *4CL*, the remaining genes exhibit poor collinearity, possibly due to gene changes during evolution (Fig. 3B, C). Our identification of key gene families in the chlorogenic acid synthesis pathway revealed the absence of the *HQT* and *HCGQT* genes required for routes 2, 3, and 4 in ramie (Fig. 3A). We constructed separate phylogenetic trees for the five genes *PALs, C4Hs, 4CLs, C3Hs*, and *HCTs* found in ramie. The results indicate that all these genes originated in terrestrial plants, and *PALs* and *4CLs* diverged within angiosperms, leading to the inability of some plants to synthesize chlorogenic acid (Fig. S7).

Interestingly, in the gap-free genome, we observed an expansion of chlorogenic acid biosynthesis key genes, particularly the *C4H* gene. Ramie harbors a total of six *C4H* genes, five of which belong to the expanded group. This may be one reason for ramie’s high chlorogenic acid content (Fig. 3B).

### Comparing metabolism in ramie roots and leaves

To explore metabolic variation between ramie leaves and roots, we performed metabolomic analysis using LC-MS/MS. The results showed that 903 and 396 compounds were obtained from characterizing both positive (POS) and negative (NEG) modes, respectively. Of these compounds, 27.14% and 38.39% were annotated as lipids and lipid-like molecules in the leaf and root, respectively. Meanwhile, phenylpropanoids and polyketides accounted for 18.74% and 19.05%, respectively, while organic acids and derivatives accounted for 12.89% and 11.90%, respectively, in metabolism (Fig.  S8). The metabolites detected based on POS and NEG modes were assigned to 14 and 13 KEGG pathways, respectively (Fig.  S9).

**Figure 4:**
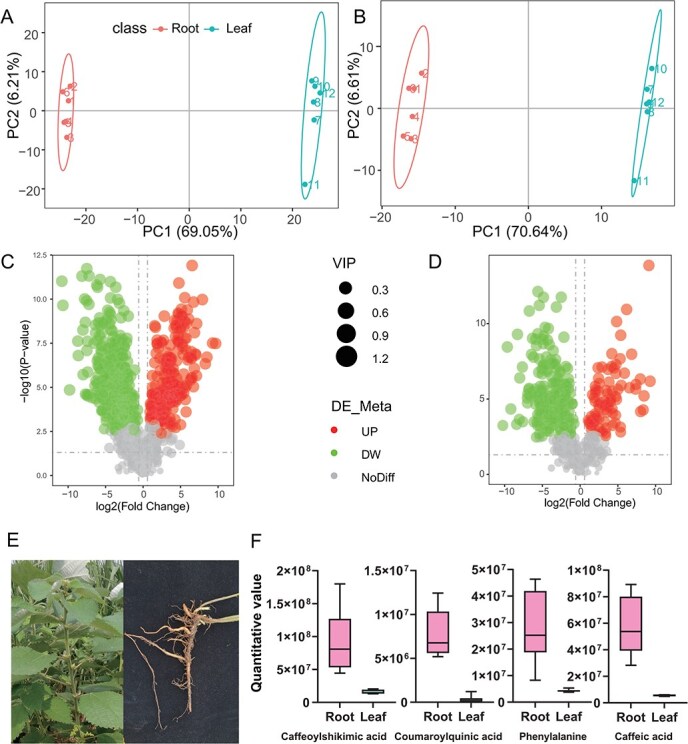
The key metabolites of the chlorogenic acid metabolic pathway in ramie. (A–B) OPLS-DA scores of leaf and root metabolomes (A, POS mode; B, NEG mode). (C–D) Volcano plots of metabolites in ramie leaves and roots (C, POS mode; D, NEG mode). E. Ramie leaves and roots. (F) Accumulation of chlorogenic acid and its upstream metabolites in different tissues.

We then compared the metabolites in ramie leaves and roots using principal component analysis and partial least squares discriminant analysis (PLS-DA) modeling (Fig. 4A-B, Fig.  S10). The results showed that the metabolic profiles of the leaf and root could be distinguished as two groups, indicating the reproducibility and reliability of the analysis. Then, the differentially expressed metabolites (DEMs) were identified using three parameters: VIP (>1.0), fold change (FC > 1.5), and *P*-value (<0.05). This resulted in the identification of 310 up-regulated and 522 down-regulated DEMs (Fig. 4C, D). The results revealed significantly higher procyanidin A2 concentrations in root extracts than in leaf samples, with a 9.28-fold difference. Similarly, the content of tectorigenin in roots was 7.90-fold higher than in leaves. Conversely, bakuchiol content in leaves was 10.84-fold higher in roots, and levistilide A content in leaves was 7.49-fold higher than in roots. These findings support the idea that metabolites are tissue-specific (Fig.  S11).

### Biosynthetic pathway of chlorogenic acid in ramie

We observed distinct differences in the levels of chlorogenic acid, its precursor phenylalanine, and various intermediate compounds in leaves and roots. For example, the contents of caffeoyl shikimic acid, coumaroyl quinic acid, phenylalanine, caffeic acid, and chlorogenic acid were significantly higher in ramie roots than in leaves. The chlorogenic acid content in roots was 10 times higher than in leaves (Fig. 4E-F). Conversely, some intermediates in the upstream pathway of chlorogenic acid biosynthesis, such as p-coumaroyl shikimic acid, p-coumaric acid, and caffeoyl glucose, exhibited higher concentrations in leaves than in roots. This suggests that the rate at which the substrate is consumed for chlorogenic acid biosynthesis is lower in leaves than in roots (Fig.  S12).

**Figure 5:**
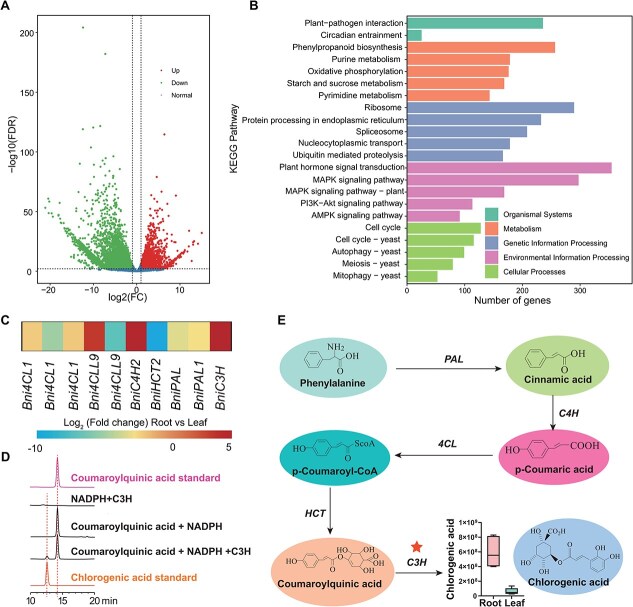
Metabolic pathway and differential gene identification of chlorogenic acid in ramie. (A) Volcano map of differential genes in ramie leaves and roots. (B) KEGG enrichment analysis of differential genes. (C) Identification of the differential genes of the chlorogenic acid metabolic pathway in ramie leaves and roots. (D) C3H Protease Activity Catalysis. (E) The chlorogenic acid synthesis pathway in ramie, the shaded ellipses in different colors represent key metabolites identified in the ramie metabolome.

To explore genes potentially involved in the biosynthesis of chlorogenic acid, we performed RNA sequencing and compared the expression profiles of roots and leaves. A total of 7239 differentially expressed genes (DEGs) were identified between the two tissues (Fig. 5A; Fig.  S13). Six DEGs were randomly selected for quantitative reverse transcription PCR (qRT-PCR) analysis (Table S10, Fig. S14), which confirmed the reliability of the RNA-seq data. More than 200 DEGs were enriched in the ‘Phenylpropanoid biosynthesis’ pathway (4.38%) (Fig. 5B), indicating differences in the phenylpropanoid pathway between the two tissues investigated. Ten of these DEGs encode essential enzymes for chlorogenic acid biosynthesis, including two *PAL* genes, five *4CL* or *4CL*-like genes, one *C4H* gene, one *HCT* gene, and one *C3H* (Fig. 5C). We aligned 7239 differentially expressed genes with transcription factors from the Plant Transcription Factor Database (PlantTFDB: https://planttfdb.gao-lab.org/), identifying 4487 differentially expressed transcription factor genes. Of these, 162 belonged to the MYB family, 288 to the NAC family, 189 to the WRKY family, and 464 to the bHLH family. We randomly selected 10 transcription factors and analyzed the differences in their expression between leaves and roots. The results showed that the vast majority of transcription factors exhibited higher expression levels in roots than in leaves, which is consistent with the patterns of chlorogenic acid content (Table S11).

Notably, the expression trends of some genes do not consistently correlate with metabolites. For example, *HCT2*, *4CL1*, and one of the *4CLL9* are more highly expressed in leaves, whereas CGA accumulates more in roots. This discrepancy may arise because the accumulation of end products potentially inhibits key enzyme activity, necessitating dynamic regulation to mitigate this inhibition [29]. Alternatively, organelle compartmentalization may restrict the diffusion of intermediates, thereby preventing toxic accumulation and regulating reaction rates via local concentration gradients. These findings reflect the complexity of gene-to-protein expression pathways [30]. Interestingly, we found that the expression levels of the *Bni4CL9, BniC4H*, and *BniC3H* genes were significantly higher in roots than in leaves, whereas *BniPAL and BniHCT2* expression was lower in roots than in leaves, and the chlorogenic acid content was significantly higher in roots than in leaves (Fig. 5C, E). To verify that route 1 is the primary pathway for chlorogenic acid synthesis in ramie, we measured the C3H protease activity. The results further confirmed that the C3H protein catalyzes the conversion of coumaroylquinic acid (a chlorogenic acid precursor) into chlorogenic acid (Fig. 5D). Based on these findings, we speculated that route 1 may represent the primary route for chlorogenic acid synthesis in ramie (Fig. 5E).

## Discussion

In recent years, more crops have completed genome sequencing, especially the T2T genome, which has greatly improved the identification of key crop traits [31–33]. However, the complete genome of ramie has not been reported. In this study, the complete genome of Ramie, containing 14 chromosomes with a size of 344.21 Mb, was constructed through the optimization of various strategies, which exceeded previous reports ‘*Bniv*_ZSZ1’ and ‘*Bniv*_QY’ genomes, and the number of identified genes climbed from 20 693 to 25 853. The length of genome N50 in this version reached 24.4 Mb, which was greatly improved from the previous genome versions [16, 17, 24]. The results of comparative analysis demonstrated that the genomes of the three ramie varieties have structural variants such as inversions and translocations. It is noteworthy that in Ramie, nine of the chromosomes had miters located at the ends of the chromosomes (Fig. 1C), which is consistent with the results of previous studies [34]. These new findings serve as a theoretical basis for the study of the genetic function of Ramie. In addition, our genome expansion genes include a large number of auxin-related genes, homologs of which are found in most plants, indicating that the functions of auxin-related genes are highly conserved throughout plant evolution [35].

Ramie is a perennial bast fiber crop native to China with rich germplasm resources for textile and paper making. Plant fibers were mainly composed of cellulose, lignin, and hemicellulose, of which lignin was the second most abundant natural compound deposited in specific cell walls of higher plants and is one of the products of phenylalanine pathway biosynthesis [36]. Interestingly, chlorogenic acid is also a metabolite of the phenylalanine pathway and its chemical structure is very similar to that of some lignin intermediates [37, 38], which may be the main reason for the correlation between the accumulation of chlorogenic acid and the deposition of lignin in ramie [39, 40].

Plants were a rich source of valuable compounds used in traditional medicines, pharmaceuticals, and agricultural chemicals. As modern biotechnology and enzyme chemistry had developed, the biosynthetic pathways of chlorogenic acid had gradually been elucidated [2]. Researchers had recently discovered that enzymes responsible for the biosynthesis of specialized metabolites cluster in specific genomic regions known as biosynthetic gene clusters (BGCs) [41]. BGCs have been widely reported in plants and are involved in the synthesis of various metabolites [42]. However, in our study, the genes involved in chlorogenic acid synthesis did not exhibit the characteristics of gene clusters. We speculate that the absence of clustering may stem from the fact that CGA biosynthesis belongs to the ubiquitous phenylpropanoid metabolic pathway, which is typically dispersed throughout the genome rather than clustered together [37].

Recently, chlorogenic acid was identified as the primary pharmacological component of ramie; however, the molecular mechanisms underlying its accumulation remain unclear [10]. The metabolome showed that the chlorogenic acid content in ramie roots exhibited significantly higher levels than leaves (Fig. 5D). Several structural genes in the chlorogenic acid synthesis and metabolism pathway were found to have multiple copies in the whole genome, but the transcriptome data showed that some of these genes were not transcribed (Fig. 5C). It was possible that the functions of the multicopy genes were redundant in chlorogenic acid synthesis in Ramie. Some genes maintain high transcript levels in multiple copies, suggested that they could be a key factor in these replications. 4CL was a key rate-limiting enzyme in the phenylalanine synthesis pathway and provided substrates for the action of the downstream phenylalanine metabolism pathway and the flavonoid metabolism pathway. In *Gossypium hirsutum*, overexpression of *Gh4CL3* increased *4CL* activity, enhanced phenolic precursors, like cinnamic acid and p-coumaric acid, and promoted chlorogenic acid synthesis [43]. We identified five *4CL* or *4CL*-like genes in the transcriptome, only one of which was highly expressed in roots, probably because the functions of the other copies of the gene were redundant.

The accumulation of chlorogenic acid in ramie roots is significantly higher than in leaves. This tissue-specific distribution pattern suggests it may serve multiple physiological and ecological functions during the plant’s adaptation to underground environments. Combined with differences in intermediate metabolite profiles revealed by metabolomics data, it is speculated that the root enrichment of chlorogenic acid represents an adaptive strategy evolved by ramie over long-term evolution. Previous studies indicate that chlorogenic acid, due to its phenolic structure, exhibits broad-spectrum antibacterial activity by inhibiting pathogen cell membrane integrity or disrupting metabolic enzyme activity [44]. Ramie may establish a chemical defense barrier through high-concentration chlorogenic acid accumulation in roots, thereby reducing the incidence of diseases such as root rot.

Here, we assembled the first gap-free T2T genome of ramie and provided detailed genomic information. Comparative genomes were identified to Ramie expansion and contraction genes, and differences from the previously assembled version of the genome were found. The metabolomes of Ramie leaves and roots were determined, and the ramie chlorogenic acid synthesis pathway was characterized. Gene expression was analyzed for possible mechanisms of chlorogenic acid synthesis regulation. Future research will explore pathways to enhance chlorogenic acid content in ramie breeding through targeted CGA enhancement via genetic engineering or metabolic engineering, laying the foundation for accelerating genetic improvement in ramie.

## Materials and methods

### Plant materials and sequencing

In the National Ramie Germplasm Resource Nursery, we selected a variety of ramie (No. 1380–2) as the material for genome sequencing. The specific method was to take the young leaves of ramie, freeze them in liquid nitrogen, and then extract high-quality DNA using the CTAB method, and then use the ultra-micro-UV spectrophotometer to detect the OD value, in which the OD260/OD280 = 1.8 ~ 2.0, which indicated that the DNA was purer. Purity, and 1.25% agarose gel electrophoresis was detected with a single band and no obvious trailing degradation, indicating better DNA integrity.

For HiFi sequencing, the DNA was then randomly fragmented into pieces (15–18 kb) by Covaris ultrasonic crusher; the large fragments were processed for enrichment and purification by magnetic beads; the fragmented DNA was submitted to damage repair and end repair; the DNA fragments were connected to stem-loop sequencing junctions at both ends, and exonuclease was employed to eliminate the fragments that cannot be connected. The constructed libraries were sequenced by PacBio Sequel II/PacBio Sequel IIe platform. For ONT Ultra-Long Sequencing‌, libraries were prepared using the SQK-ULK001 kit (Oxford Nanopore Technologies, UK) following the manufacturer’s protocol and sequenced on a PromethION platform. For ‌Hi-C Sequencing‌, Hi-C libraries were constructed from fresh young leaves using the NEBNext Ultra II DNA Library Prep Kit (NEB, USA) with DpnII digestion and sequenced on an Illumina NovaSeq 6000. ‌For Short-Read Sequencing (DNBSEQ-T7)‌, whole-genome libraries were prepared and sequenced on the DNBSEQ-T7 platform (BGI). Leaf and root tissues were snap-frozen in liquid nitrogen for subsequent transcriptomic and metabolomic analyses.

### Genome assembly and pseudochromosome construction

In the genome survey, genome size and heterozygosity were scored using Jellyfish (2.2.10) and Genomescope 2.0 with a parameter of kmer = 21 and a Kmer coverage threshold of 10 000 000 [45, 46]. HiFi reads were assembled by the default parameters of the Hifiasm software [47, 48]. Filtering reads obtained from ONT ultra along sequencing, we performed primary genome assembly of ONT ultra-long reads using NextDenovo (https://github.com/Nextomics/ NextDenovo), and to further improve single-base accuracy, we utilized NextPolish (https://github.com/Nextomics/Next Polish) for sequence optimization [49]. Subsequently, we assembled a combination of HiFi reads and ONT reads using the default parameters of the Hifiasm software [47], compared mitochondrial and chloroplast data using the blast software to remove sequences with >50% base pairs, removed bacterial contamination by BLAST RefSeq library, then removed low read-supported contigs [50].

Using ALLHiC software, which revealed chromatin interactions in three-dimensional space, sequences were clustered into different chromosome groups [51]. Manual sorting and localization were performed using Juicebox software [52]. A genome containing 14 chromosomes was obtained. Finally, we used the TGS-GapCloser software [21] to patch gaps in the genome to obtain the final version of the genome.

### Identification of telomeres and prediction of centromere regions

Telomeres were structurally conserved, and in plant genomes, a seven-base telomeric repeat sequence (CCCTAAAA at the 5′ or TTTAGGG at the 3′) was commonly used to identify the telomeric region by comparison with the genome. Based on this, we used the quartTeT toolkit to perform telomere prediction on our genome, resulting in 28 complete telomeres. Similarly, Centromeres were composed of highly repetitive sequences and a small number of genes, and we used the quartTeT toolkit to identify 14 Centromeres [22].

We used a variety of methods to assess the quality of the assembled genomes. First, N50 sequence lengths were used to show genome continuity and assess genome accuracy. Genome integrity was then assessed by BUSCO software using the viridiplantae_odb10 database [53]. In addition, Illumina reads were mapped using BWA-MEM [54]; The HiFi and ONT sequencing data were aligned to the reference genome using minimap2 [55], followed by LAI index computation for genome assembly evaluation [56].

### Genome annotation

The genome was first annotated for repetitive sequences using RepeatModeler and EDTA [57], and the repetitive regions of the genome were soft-masked using RepeatMasker and subsequently integrated using LTR_retriever [58]. Then the gene coding regions were predicted by *de novo* prediction and homology by combining August, GlimmerHMM, Geneid, SNAP, and GeMoMa. For *de novo* prediction dataset, which was acquired by leveraging Augustus [59]. Based on homology-based gene prediction, we chose *Arabidopsis thaliana* and two previous ramie varieties using GeMoMa [60]. High-confidence gene structures were constructed by EVidenceModeler through weighted consensus of multiple prediction methods [61]. Finally, the assembled genome sequences were integrated using PASA software [62].

Genomic annotation integrity was assessed using BUSCO software [53]. The longest protein sequences of the genes were obtained as representative sequences of the genes for functional annotation, which were annotated using the emapper process provided by eggNOG, and compared with the UniProt, Pfam, and InterPro protein databases to determine the biological functions of the genes. The tRNA in the genome was identified based on its structural features and rRNA was predicted by the rRNA database.

### Genome comparison and variation identification

Genome comparisons were performed using Nucmer (v4.0.0rc1) for *Bni_1380* and two additional varieties (‘*Bniv_*ZSZ1’ and ‘*Bniv_*QY’) [63]. The results were provided to the SyRI pipeline, which utilized them to identify colinear blocks, structural variations (insertions, deletions, duplications, translocations, and inversions), and sequence divergences [64].

### Gene family cluster analysis

Together with *Boehmeria nivea*1380, *Arabidopsis thaliana*, *Oryza sativa*, *Solanum* lyc*opersicum*, *Vitis vinifera, Theobroma cacao, Camelina sativa, Fagaria vesca, Ziziphus jujuba, Helianthus annuus, Morus notabilis, Boehmeria nivea* ZSZ, *Boehmeria nivea* QY, and *Coffea arabica*, 14 plants were analyzed for genome evolution. All amino acid sequences of the species were clustered into gene families via OrthoFinder software.

### Phylogenetic tree construction

Used single-copy genes, an interspecies phylogenetic tree was constructed using the RAxML model (https://github.com/amkozlov/raxml-ng/wiki/Input-data-#evolutionary-model), followed by timetree (http://timetree.org/) to add fossil time points, and finally, the time of species divergence was estimated by way of the mcmctree program in the PAML package.

### Gene family contraction and expansion

Drawing from the number of gene families in different species, the cafe5 software was used to infer the size of gene families in ancestral species by simulating the evolutionary rate of gene families on a phylogenetic tree, using birth and death processes to simulate the gain and loss of genes in the user-specified phylogenetic tree [65]. *P* < 0.05 was defined as a significant amplification or a significant contraction, and gene families that were significantly amplified or significantly contracted were subjected to functional enrichment analysis.

### Collinearity analysis

We compared all gene sequences of three different varieties of ramie using Blast and subsequently analyzed these results for collinearity using MCScanX to ascertain similar gene pairs. Chromosomal locations of syntenic genes were determined by JCVI software, enabling covariate block identification.

### RNA sequencing

Leaves and roots were sequenced for RNA using three individuals as three replicates. Following RNA extraction, Illumina-compatible libraries were generated with NEBNext UltraTM reagents (300 bp fragment size). Library sequencing was conducted on an Illumina platform with HiSeq PE Cluster Kit v4 cBot, adhering to the manufacturer’s technical manual. Quality-filtered reads were mapped to the reference genome using HISAT2 (default settings). Gene expression levels were calculated as FPKM values, with differential expression analysis performed in DESeq2 (|log2FC| ≥1, *P* < 0.05).

### Transcription factor identification

Transcription factor identification of differentially expressed genes was performed using the BLAST tool. We aligned the protein sequences of 7239 differentially expressed genes against transcription factors in the Plant Transcription Factor Database (PlantTFDB: https://planttfdb.gao-lab.org/) [66].

### LC-MS–based untargeted metabolomic analysis

Six individuals of leaf and root were used as six replicate samples for metabolomic analysis. Approximately 60 mg of tissue-extracted metabolites were used for each sample. Separation was performed on an HSS T3 C18 column (100 mm × 2.1 mm × 1.8 μm, Waters Corporation), and the column temperature was maintained at 45°C. LC-MS metabolic profiling was performed using an ACQUITY UHPLC system (Waters Corporation, Milford, USA) and an AB SCIEX Triple TOF 5600 system (AB SCIEX Triple). TOF 5600 system (AB SCIEX, Framingham, MA, USA) in ESI positive and negative ion modes with the following parameters: spray voltage, 3800 V; capillary temperature, 320°C; auxiliary gas heater temperature, 350°C; sheath gas flow rate, 35 Arb; auxiliary gas flow rate, eight Arb; S-lens RF level, 50; mass range, mass range m 50; mass range, mass range m/z; full millisecond resolution, 70 000; MS/MS resolution, 17 500; NCE/step NCE, 10, 20, 40. Compound annotation was performed based on RT-m/z correlation and MS/MS spectral matching using HMDB and LIPID MAPS reference databases.

### Assay for C3H protein activity in ramie

The C3H protein activity assay system comprised 0.2 mM coumaroylquinic acid, 1 mM NADPH, and 400–600 μg/ml of proteins from ramie tissue, dissolved in a 100 mM sodium phosphate buffer at pH 7.4. The reaction was incubated at 30°C for 2 hours, after which it was terminated using 10% (v/v) acetic acid at one-tenth of the reaction volume. The reaction products were analyzed using high-performance liquid chromatography (HPLC) with a photodiode array detector (250–500 nm) and the methodology previously described for C3H reaction products was followed. Equal volumes of the test samples were placed in 0.3 ml autosampler vials and analyzed by HPLC using a Gemini 5 μm C6-Phenyl 110 A column (Phenomenex; 250 × 3.0 mm × 5 μm) under a dual-solvent system (solvent A: deionized water containing 0.0125% [v/v] trifluoroacetic acid; solvent B: acetonitrile containing 0.0125% [v/v] trifluoroacetic acid) at a flow rate of 1 ml·min^−1^. The HPLC conditions were set as follows: 5-minute isocratic elution at 10% solvent B; gradient elution to 25% solvent B over 30 minutes; isocratic elution at 25% solvent B for 3 minutes; gradient elution to 100% solvent B over 1 minute; isocratic elution at 100% solvent B for 4 minutes; gradient elution to 10% solvent B over 5 minutes; and finally, isocratic equilibration at 10% solvent B for 5 minutes [67]. Negative controls included NADPH + C3H, coumaroylquinic acid + NADPH, and coumaroylquinic acid + NADPH + C3H. All treatments were performed under identical experimental conditions with three biological replicates.

### Real-time fluorescent quantitative PCR

Based on the transcriptome sequencing results, we randomly selected six DEGs from all the differential genes. We found the corresponding nucleotide sequences from the genome and verified them by real-time quantitative PCR (qRT-PCR).

## Supplementary Material

Web_Material_uhag144

## Data Availability

The raw sequence data of genome sequence and RNA sequencing of leaf and root have been deposited in the Genome Sequence Archive in National Genomics Data Center (NGDC, China) (GSA: CRA021230) that are publicly accessible at https://ngdc.cncb.ac.cn/gsa.
