# Supplementary material for: The telomere-to-telomere genome and comparative analysis from algae to higher plants reveal origin and evolution of biosynthesis of chlorogenic acid pathway and production in ramie (Boehmeria nivea L.)
Source: Hortic Res. 2026 Apr 16;13(8):uhag144. doi: 10.1093/hr/uhag144 (PMC13454483; doi:10.1093/hr/uhag144)
Supplement: Supplementary file 1 [file web_material_uhag144.zip › Fig.S3.pdf]

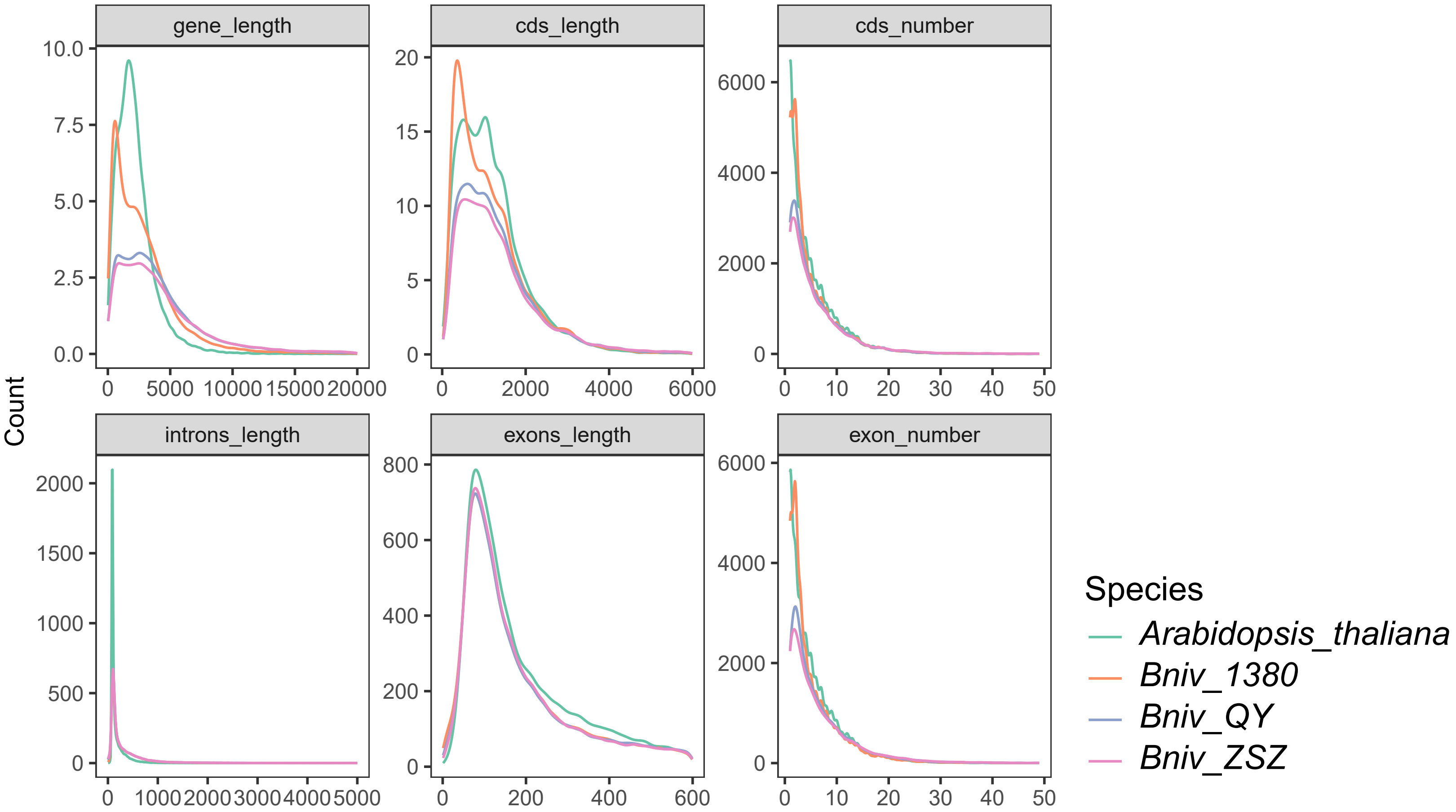

gene_length
cds_length
cds_number
introns_length
exons_length
exon_number
Count
Species
Arabidopsis_thaliana
Bniv_1380
Bniv_QY
Bniv_ZSZ
