# Supplementary material for: The telomere-to-telomere genome and comparative analysis from algae to higher plants reveal origin and evolution of biosynthesis of chlorogenic acid pathway and production in ramie (Boehmeria nivea L.)
Source: Hortic Res. 2026 Apr 16;13(8):uhag144. doi: 10.1093/hr/uhag144 (PMC13454483; doi:10.1093/hr/uhag144)
Supplement: Supplementary file 1 [file web_material_uhag144.zip › Fig.S4.pdf]

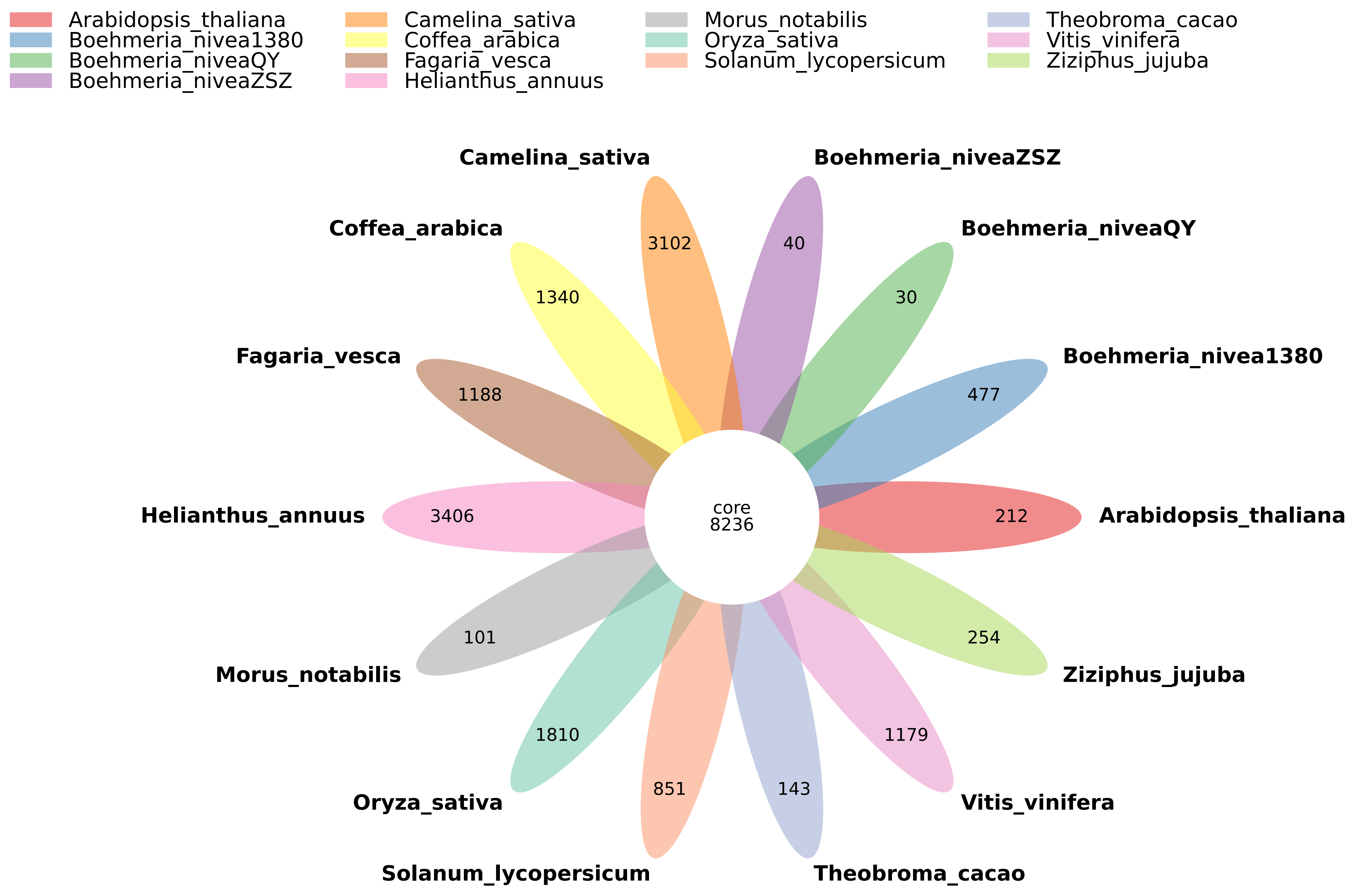
Arabidopsis_thaliana
Boehmeria_nivea1380
Boehmeria_niveaQY
Boehmeria_niveaZSZ
Camelina_sativa
Coffea_arabica
Fagaria_vesca
Helianthus_annuus
Morus_notabilis
Oryza_sativa
Solanum_lycopersicum
Theobroma_cacao
Vitis_vinifera
Ziziphus_jujuba
Camelina_sativa
3102
Boehmeria_niveaZSZ
40
Coffea_arabica
1340
Boehmeria_niveaQY
30
Fagaria_vesca
1188
Boehmeria_nivea1380
477
Helianthus_annuus
3406
core
8236
212
Arabidopsis_thaliana
101
Morus_notabilis
254
Ziziphus_jujuba
1810
Oryza_sativa
1179
Vitis_vinifera
851
Solanum_lycopersicum
143
Theobroma_cacao
