# Supplementary material for: The telomere-to-telomere genome and comparative analysis from algae to higher plants reveal origin and evolution of biosynthesis of chlorogenic acid pathway and production in ramie (Boehmeria nivea L.)
Source: Hortic Res. 2026 Apr 16;13(8):uhag144. doi: 10.1093/hr/uhag144 (PMC13454483; doi:10.1093/hr/uhag144)
Supplement: Supplementary file 1 [file web_material_uhag144.zip › Fig.S5.pdf]

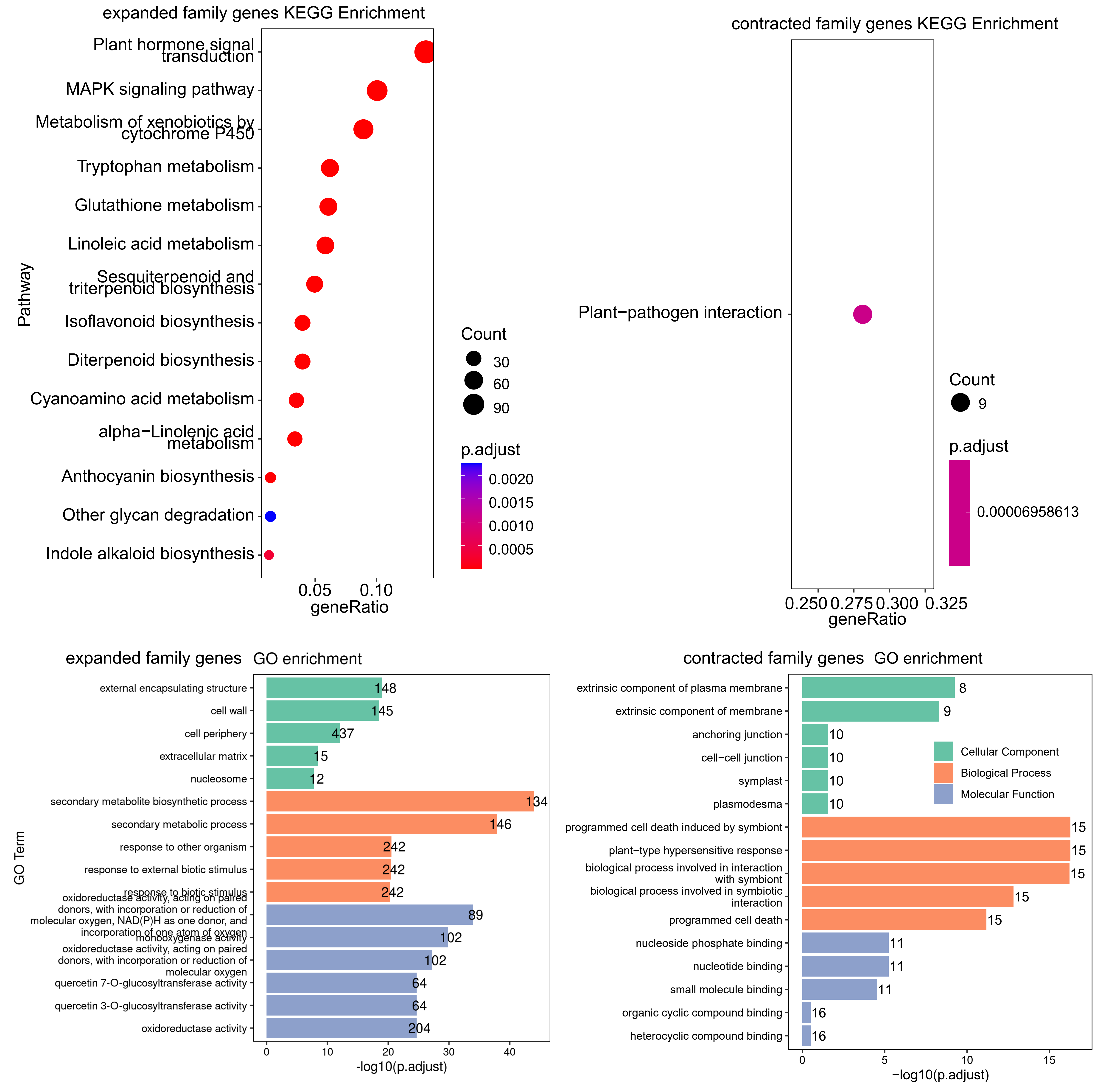
expanded family genes KEGG Enrichment
Plant hormone signal transduction
MAPK signaling pathway
Metabolism of xenobiotics by cytochrome P450
Tryptophan metabolism
Glutathione metabolism
Linoleic acid metabolism
Sesquiterpenoid and triterpenoid biosynthesis
Isoflavonoid biosynthesis
Diterpenoid biosynthesis
Cyanoamino acid metabolism
alpha−Linolenic acid metabolism
Anthocyanin biosynthesis
Other glycan degradation
Indole alkaloid biosynthesis
Pathway
0.05
0.10
geneRatio
Count
30
60
90
p.adjust
0.0020
0.0015
0.0010
0.0005
contracted family genes KEGG Enrichment
Plant−pathogen interaction
0.250
0.275
0.300
0.325
geneRatio
Count
9
p.adjust
0.00006958613
expanded family genes GO enrichment
external encapsulating structure
cell wall
cell periphery
extracellular matrix
nucleosome
secondary metabolite biosynthetic process
secondary metabolic process
response to other organism
response to external biotic stimulus
response to biotic stimulus
oxidoreductase activity, acting on paired donors, with incorporation or reduction of molecular oxygen, NAD(P)H as one donor, and incorporation of one atom of oxygen
monooxygenase activity
oxidoreductase activity, acting on paired donors, with incorporation or reduction of molecular oxygen
quercetin 7-O-glucosyltransferase activity
quercetin 3-O-glucosyltransferase activity
oxidoreductase activity
148
145
437
15
12
134
146
242
242
242
89
102
102
64
64
204
GO Term
0
10
20
30
40
-log10(p.adjust)
contracted family genes GO enrichment
extrinsic component of plasma membrane
extrinsic component of membrane
anchoring junction
cell−cell junction
symplast
plasmodesma
programmed cell death induced by symbiont
plant−type hypersensitive response
biological process involved in interaction with symbiont
biological process involved in symbiotic interaction
programmed cell death
nucleoside phosphate binding
nucleotide binding
small molecule binding
organic cyclic compound binding
heterocyclic compound binding
8
9
10
10
10
10
15
15
15
15
15
11
11
11
16
16
Cellular Component
Biological Process
Molecular Function
0
5
10
15
−log10(p.adjust)
