# Supplementary material for: The telomere-to-telomere genome and comparative analysis from algae to higher plants reveal origin and evolution of biosynthesis of chlorogenic acid pathway and production in ramie (Boehmeria nivea L.)
Source: Hortic Res. 2026 Apr 16;13(8):uhag144. doi: 10.1093/hr/uhag144 (PMC13454483; doi:10.1093/hr/uhag144)
Supplement: Supplementary file 1 [file web_material_uhag144.zip › Fig.S6.pdf]

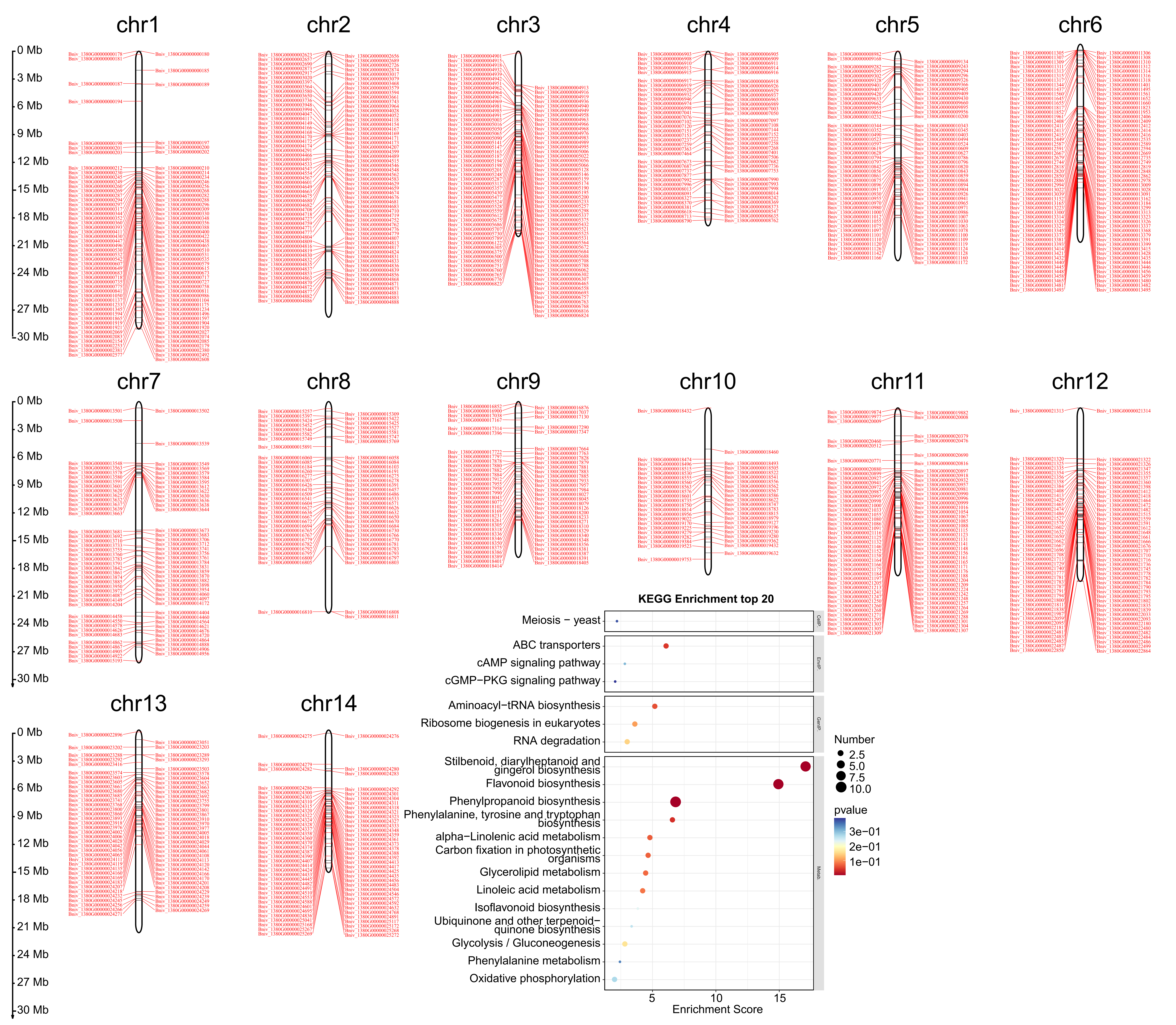

chr1
chr2
chr3
chr4
chr5
chr6
chr7
chr8
chr9
chr10
chr11
chr12
chr13
chr14
0 Mb
3 Mb
6 Mb
9 Mb
12 Mb
15 Mb
18 Mb
21 Mb
24 Mb
27 Mb
30 Mb
KEGG Enrichment top 20
Meiosis − yeast
ABC transporters
cAMP signaling pathway
cGMP−PKG signaling pathway
Aminoacyl−tRNA biosynthesis
Ribosome biogenesis in eukaryotes
RNA degradation
Stilbenoid, diarylheptanoid and gingerol biosynthesis
Flavonoid biosynthesis
Phenylpropanoid biosynthesis
Phenylalanine, tyrosine and tryptophan biosynthesis
alpha−Linolenic acid metabolism
Carbon fixation in photosynthetic organisms
Glycerolipid metabolism
Linoleic acid metabolism
Isoflavonoid biosynthesis
Ubiquinone and other terpenoid−quinone biosynthesis
Glycolysis / Gluconeogenesis
Phenylalanine metabolism
Oxidative phosphorylation
5
10
15
Enrichment Score
Number
2.5
5.0
7.5
10.0
pvalue
3e−01
2e−01
1e−01
