# Supplementary material for: The telomere-to-telomere genome and comparative analysis from algae to higher plants reveal origin and evolution of biosynthesis of chlorogenic acid pathway and production in ramie (Boehmeria nivea L.)
Source: Hortic Res. 2026 Apr 16;13(8):uhag144. doi: 10.1093/hr/uhag144 (PMC13454483; doi:10.1093/hr/uhag144)
Supplement: Supplementary file 1 [file web_material_uhag144.zip › Fig.S7.pdf]

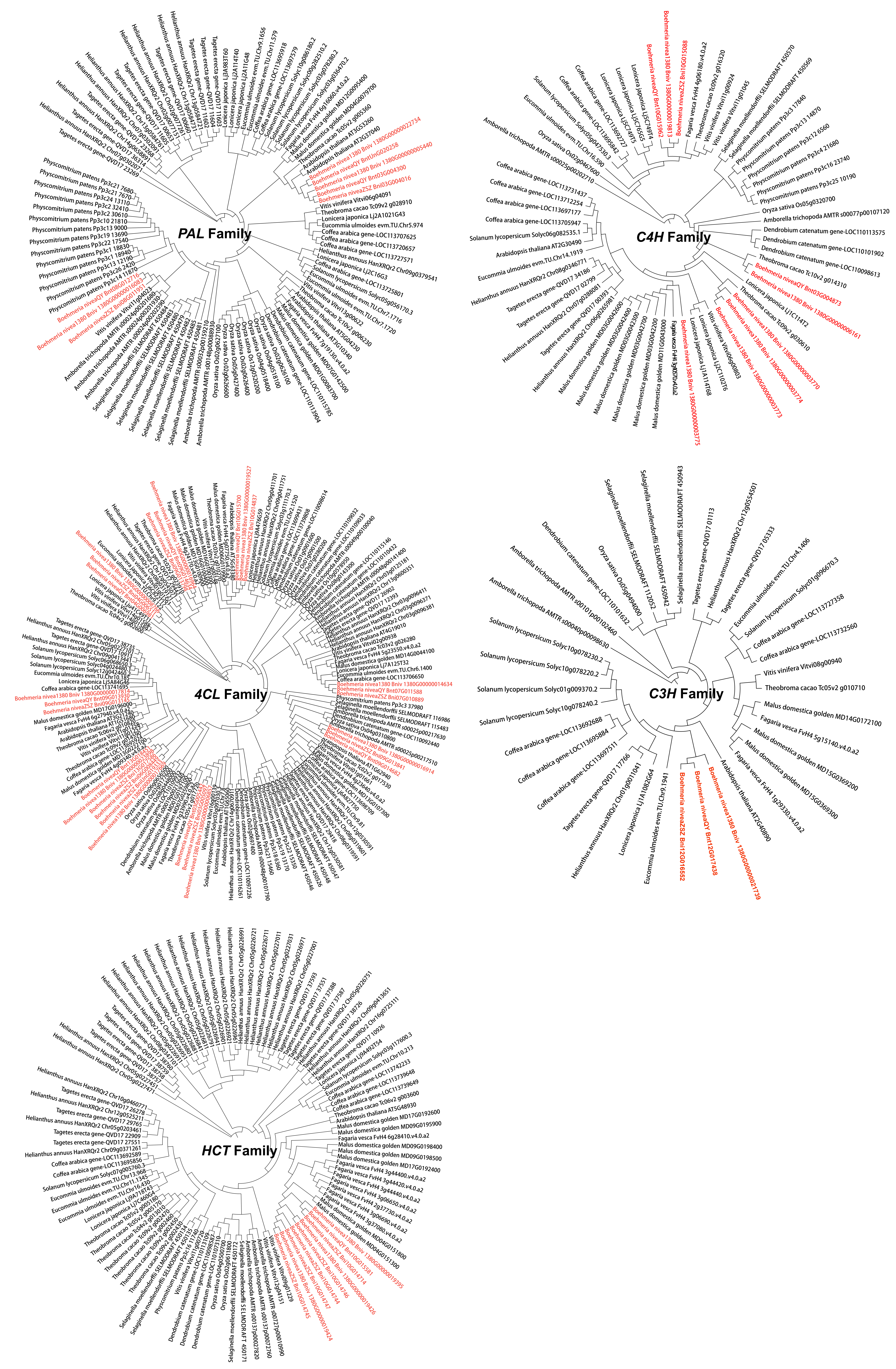

PAL Family
C4H Family
4CL Family
C3H Family
HCT Family
Selaginella moellendorffii SELMODRAFT 450943
Selaginella moellendorffii SELMODRAFT 112052
Selaginella moellendorffii SELMODRAFT 450942
Oryza sativa Os05g0494000
Dendrobium catenatum gene-LOC110101632
Amborella trichopoda AMTR s00101p00102460
Amborella trichopoda AMTR s00040p00098630
Solanum lycopersicum Solyc10g078230.2
Solanum lycopersicum Solyc10g078220.2
Solanum lycopersicum Solyc01g009370.2
Solanum lycopersicum Solyc10g078240.2
Coffea arabica gene-LOC113692688
Coffea arabica gene-LOC113695884
Coffea arabica gene-LOC113697511
Tagetes erecta gene-QVD17 17766
Helianthus annuus HanXRQr2 Chr01g0011041
Lonicera japonica Lj1A1082G64
Eucommia ulmoides evm.TU.Chr9.1941
Boehmeria niveaZSZ Bni12G016552
Boehmeria niveaQY Bnt12G017438
Boehmeria nivea1380 Bniv 1380G00000021739
Arabidopsis thaliana AT2G40890
Fagaria vesca FvH4 1g29330.v4.0.a2
Malus domestica golden MD15G0369300
Malus domestica golden MD15G0369200
Fagaria vesca FvH4 5g15140.v4.0.a2
Malus domestica golden MD14G0172100
Theobroma cacao Tc05v2 g010710
Vitis vinifera Vitvi08g00940
Coffea arabica gene-LOC113732560
Coffea arabica gene-LOC113727358
Solanum lycopersicum Solyc01g096670.3
Eucommia ulmoides evm.TU.Chr4.1406
Tagetes erecta gene-QVD17 05333
Helianthus annuus HanXRQr2 Chr12g0554501
Tagetes erecta gene-QVD17 01113
