# Supplementary material for: The telomere-to-telomere genome and comparative analysis from algae to higher plants reveal origin and evolution of biosynthesis of chlorogenic acid pathway and production in ramie (Boehmeria nivea L.)
Source: Hortic Res. 2026 Apr 16;13(8):uhag144. doi: 10.1093/hr/uhag144 (PMC13454483; doi:10.1093/hr/uhag144)
Supplement: Supplementary file 1 [file web_material_uhag144.zip › Fig.S8.pdf]

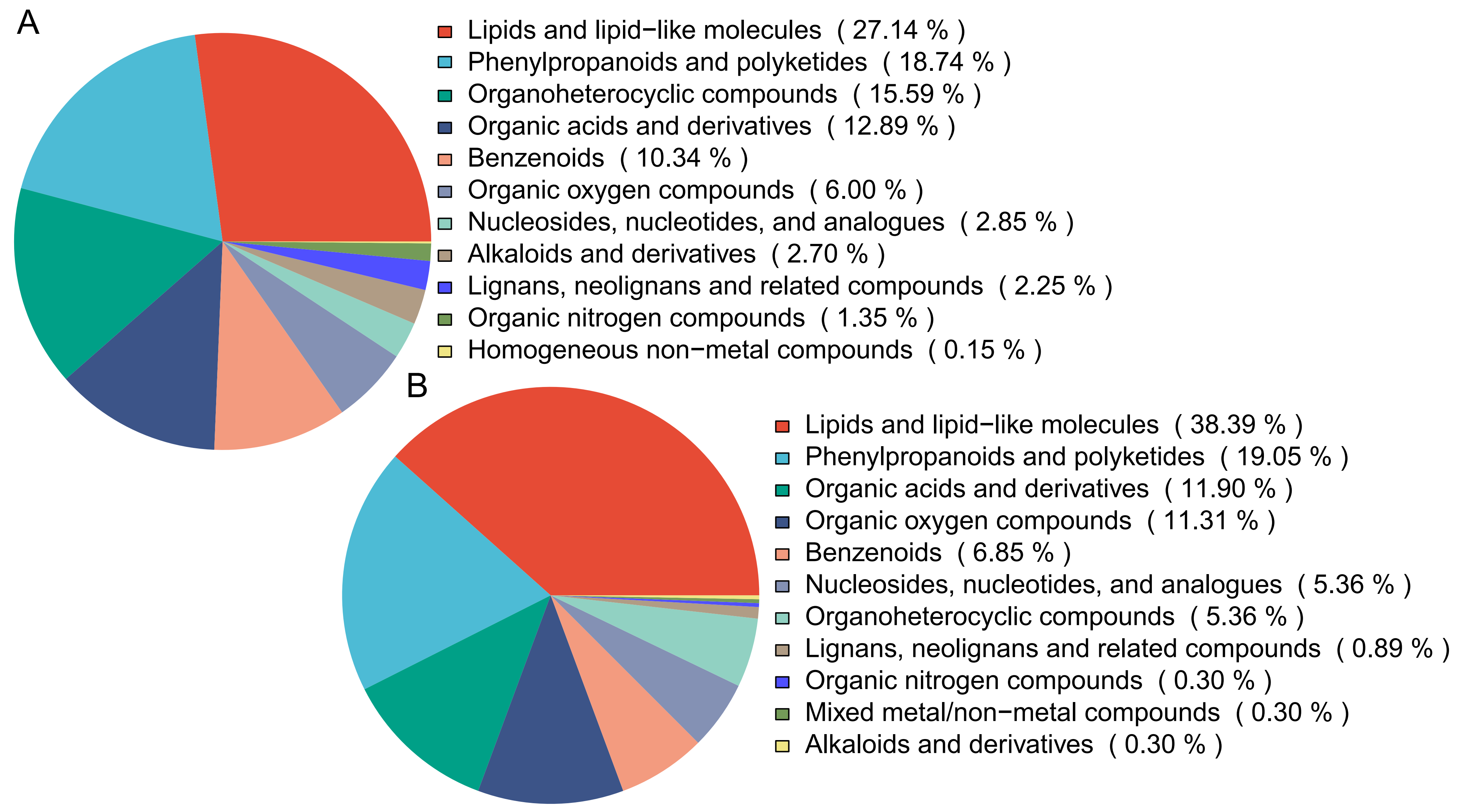
A
Lipids and lipid−like molecules (27.14 %)
Phenylpropanoids and polyketides (18.74 %)
Organoheterocyclic compounds (15.59 %)
Organic acids and derivatives (12.89 %)
Benzenoids (10.34 %)
Organic oxygen compounds (6.00 %)
Nucleosides, nucleotides, and analogues (2.85 %)
Alkaloids and derivatives (2.70 %)
Lignans, neolignans and related compounds (2.25 %)
Organic nitrogen compounds (1.35 %)
Homogeneous non−metal compounds (0.15 %)
B
Lipids and lipid−like molecules (38.39 %)
Phenylpropanoids and polyketides (19.05 %)
Organic acids and derivatives (11.90 %)
Organic oxygen compounds (11.31 %)
Benzenoids (6.85 %)
Nucleosides, nucleotides, and analogues (5.36 %)
Organoheterocyclic compounds (5.36 %)
Lignans, neolignans and related compounds (0.89 %)
Organic nitrogen compounds (0.30 %)
Mixed metal/non−metal compounds (0.30 %)
Alkaloids and derivatives (0.30 %)
