# Supplementary material for: The telomere-to-telomere genome and comparative analysis from algae to higher plants reveal origin and evolution of biosynthesis of chlorogenic acid pathway and production in ramie (Boehmeria nivea L.)
Source: Hortic Res. 2026 Apr 16;13(8):uhag144. doi: 10.1093/hr/uhag144 (PMC13454483; doi:10.1093/hr/uhag144)
Supplement: Supplementary file 1 [file web_material_uhag144.zip › Supplementary Figure.docx]

**The telomere-to-telomere genome and comparative analysis from algae to higher plants reveal origin and evolution of biosynthesis of chlorogenic acid pathway and production in ramie (*Boehmeria nivea* L.)**

Jianrong Chen^1,#^, Qian Lin^2,3,#^, Yangyu Tao^4, #^, Yuwei Guo^4^, Jie Guo^4^, Hangfan Niu^4^, Xintong Zhou^4^, Yifan Ding^4^, Fu Li^4^, Touming Liu^4^, Yanzhou Wang^3^, Song Gao^4^

^1^ School of Biological and Chemical Engineering, Changsha University, Changsha, China

^2^ Hunan Provincial Key Laboratory of the Traditional Chinese Medicine Agricultural Biogenomics, Changsha Medical University, Changsha, Hunan, China

^3^ Institute of Bast Fiber Crops, Chinese Academy of Agricultural Sciences, Changsha, China

^4^ Key Laboratory of Biobreeding for Specialty Horticultural Crops of Jiangsu Province, College of Horticulture and Landscape Architecture, Yangzhou University, Yangzhou, China

^#^ These authors contributed equally to this article

Correspondence should be addressed to Song Gao ([gaosong@yzu.edu.cn](mailto:gaosong@yzu.edu.cn)); Yanzhou Wang ([wyzhcf@163.com](mailto:wyzhcf@163.com)).

**Fig. S1 Plot showing heterozygosity resulting from k-mer analysis.**

**Fig.** **S2 BUSCO evaluation base on viridiplantae_odb10, eudicots_odb10 and embryophyta_odb10.**

**Fig.** **S3** **All species gene structure statistics.**

**Fig.** **S4 Flower plot of 14 species.**

**Fig.** **S5 KEGG and GO functional enrichment analysis of expanded family genes and contracted family genes observed in *Bniv_1380*.**

**Fig.** **S6 Distribution of specific genes in the genome *Bniv_*1380 and KEGG functional enrichment analysis of unique genes of *Bniv_*1380.**

**Fig.** **S7 Phylogenetic trees of *PALs, C4Hs, 4CLs, C3Hs*, and *HCTs***

**Fig.** **S8 Distribution of metabolites identified at the primary level. (A) POS mode; (B) NEG mode.**

**Fig.** **S9 KEGG pathway classification: metabolites detected and annotated. (A) POS mode; (B) NEG mode. The x-axis represents the number of metabolites identified, and the y-axis represents the KEGG pathway.**

**Fig.** **S10 The OPLS-DA score plots for different comparison groups. (A) POS mode; (B) NEG mode.**

**Fig.** **S11 Accumulation of procyanidin A2, tectorigenin, bakuchiol and levistilide A content in different tissues.**

**Fig.** **S12 Accumulation of Caffeoyl glucose, Coumaric acid and p-Coumaroyl shikimic acidchlorogenic acid metabolites in different tissues.**

**Fig. S13 Heatmap of differentially expressed genes among different tissues.**

**Fig. S14 QRT-PCR was performed to validate 6 DEGs identified by RNA-seq in root and leaf of ramie.** Red lines and left axis indicate qRT-PCR results. Histogram indicates and right axis indicate the RNA-seq results. Values are mean ± SE (n = 4).
