# Supplementary material for: The telomere-to-telomere genome and comparative analysis from algae to higher plants reveal origin and evolution of biosynthesis of chlorogenic acid pathway and production in ramie (Boehmeria nivea L.)
Source: Hortic Res. 2026 Apr 16;13(8):uhag144. doi: 10.1093/hr/uhag144 (PMC13454483; doi:10.1093/hr/uhag144)
Supplement: Supplementary file 1 [file web_material_uhag144.zip › Supplementary Table.docx]

**The telomere-to-telomere genome and comparative analysis from algae to higher plants reveal origin and evolution of biosynthesis of chlorogenic acid pathway and production in ramie (*Boehmeria nivea* L.)**

Jianrong Chen^1,#^, Qian Lin^2,3,#^, Yangyu Tao^4, #^, Yuwei Guo^4^, Jie Guo^4^, Hangfan Niu^4^, Xintong Zhou^4^, Yifan Ding^4^, Fu Li^4^, Touming Liu^4^, Yanzhou Wang^3^, Song Gao^4^

^1^ School of Biological and Chemical Engineering, Changsha University, Changsha, China

^2^ Hunan Provincial Key Laboratory of the Traditional Chinese Medicine Agricultural Biogenomics, Changsha Medical University, Changsha, Hunan, China

^3^ Institute of Bast Fiber Crops, Chinese Academy of Agricultural Sciences, Changsha, China

^4^ Key Laboratory of Biobreeding for Specialty Horticultural Crops of Jiangsu Province, College of Horticulture and Landscape Architecture, Yangzhou University, Yangzhou, China

^#^ These authors contributed equally to this article

Correspondence should be addressed to Song Gao ([gaosong@yzu.edu.cn](mailto:gaosong@yzu.edu.cn)); Yanzhou Wang ([wyzhcf@163.com](mailto:wyzhcf@163.com)).

**Table S1 Data summary of k-mer analysis**

| Property | Min | Max |
| --- | --- | --- |
| Homozygous (aa) | 97.95% | 98.08% |
| Heterozygous (ab) | 1.92% | 2.05% |
| Genome Haploid Length | 347263407 bp | 350233492 bp |
| Genome Repeat Length | 179191299 bp | 180723891 bp |
| Genome Unique Length | 168072109 bp | 169509601 bp |
| Model Fit | 52.22% | 98.79% |
| Read Error Rate | 0.18% | 0.18% |

**Table S2 Data statistics of whole-genome sequencing for ‘1380’**

| Libraries | Total data (Gb) | Coverage fold |
| --- | --- | --- |
| PacBio Hifi | 8.96 | 25.6 |
| Nanopore | 54.67 | 156.2 |
| Illumina | 13.05 | 37.3 |
| Hi-C | 30.77 | 88 |
| Total | 107.45 | 307 |

**Table S3 Repeated sequences statistics for genome assembly of ‘1380’**

| Class | Count | bp Masked | % masked |
| --- | --- | --- | --- |
| CMC-EnSpm | 4674 | 1663664 | 0.45% |
| MITE | 90127 | 17439766 | 4.68% |
| MULE-MuDR | 3946 | 1879460 | 0.50% |
| PIF-Harbing | 155 | 48909 | 0.01% |
| PIF-Harbinger | 1477 | 399368 | 0.11% |
| TcMar-Pogo | 968 | 200045 | 0.05% |
| hAT-Ac | 2771 | 1132354 | 0.30% |
| hAT-Charlie | 223 | 64476 | 0.02% |
| hAT-Tag1 | 166 | 42595 | 0.01% |
| hAT-Tip100 | 724 | 309487 | 0.08% |
| LTR | -- | -- | -- |
| Cassandra | 1796 | 111183 | 0.03% |
| Caulimovirus | 504 | 260504 | 0.07% |
| Copia | 20364 | 11560495 | 3.10% |
| ERVL | 198 | 129335 | 0.03% |
| Gypsy | 75010 | 51069989 | 13.71% |
| unknown | 673 | 446922 | 0.12% |
| RC | -- | -- | -- |
| Helitron | 1319 | 932030 | 0.25% |
| Unknown | 111005 | 134450671 | 36.09% |
| total interspersed | 321253 | 224061566 | 60.15% |
| TandemRepeat | 71004 | 3870280 | 1.04% |
| Total | 392257 | 227931846 | 61.19% |

**Table S4 All gene predict statistics for genome assembly of ‘1380’**

| Category | SNAP | Glimmer HMM | GeneID | Augustus | GeneMark-ET | GeMoMa | StringTie | PASA | EVM | PASA-update | Final Set |
| --- | --- | --- | --- | --- | --- | --- | --- | --- | --- | --- | --- |
| Number of genes: | 36,355 | 11,414 | 49,064 | 32,629 | 27,940 | 18,345 | 33,629 | 87,187 | 28,910 | 29,336 | 25,853 |
| Average gene length: | 3,084.01 | 3,669.12 | 1,215.52 | 2,798.57 | 2,972.97 | 2,959.99 | 4,498.84 | 5,228.56 | 2,581.86 | 2,973.24 | 3,098.72 |
| Total number of cds: | 169,192 | 40,682 | 162,875 | 174,362 | 147,693 | 103,224 | 186,998 | 472,376 | 143,131 | 140,320 | 130,070 |
| Total cds length: | 28,403,638 | 10,452,128 | 34,526,154 | 39,228,921 | 33,577,548 | 24,290,499 | 40,564,350 | 94,459,128 | 32,659,849 | 32,962,225 | 30,852,946 |
| Average cds length: | 167.88 | 256.92 | 211.98 | 224.99 | 227.35 | 235.32 | 216.92 | 199.97 | 228.18 | 234.91 | 237.2 |
| Average number of cds per gene: | 4.65 | 3.56 | 3.32 | 5.34 | 5.29 | 5.63 | 5.56 | 5.42 | 4.95 | 4.78 | 5.03 |
| Total number of exons: | 169,192 | 40,682 | 162,875 | 174,362 | 147,693 | 103,224 | 243,997 | 689,318 | 143,131 | 143,566 | 133,113 |
| Total exon length: | 28,403,638 | 10,452,128 | 34,526,154 | 39,228,921 | 33,577,548 | 24,290,499 | 71,767,888 | 201,583,863 | 32,659,849 | 39,479,207 | 37,360,410 |
| Average exon length: | 167.88 | 256.92 | 211.98 | 224.99 | 227.35 | 235.32 | 294.13 | 292.44 | 228.18 | 274.99 | 280.67 |
| Average number of exons per gene: | 4.65 | 3.56 | 3.32 | 5.34 | 5.29 | 5.63 | 7.26 | 7.91 | 4.95 | 4.89 | 5.15 |
| Number of genes with introns: | 36,347 | 11,383 | 30,212 | 32,615 | 23,234 | 14,394 | 30,225 | 79,520 | 23,468 | 23,964 | 20,896 |
| Total number of introns: | 132,837 | 29,268 | 113,811 | 141,733 | 119,753 | 84,879 | 210,368 | 602,131 | 114,221 | 114,230 | 107,260 |
| Total intron length: | 83,715,379 | 31,427,153 | 25,112,355 | 52,085,759 | 49,487,272 | 30,010,525 | 79,523,528 | 254,278,631 | 41,981,598 | 43,948,512 | 42,750,748 |
| Average intron length: | 630.21 | 1,073.77 | 220.65 | 367.49 | 413.24 | 353.57 | 378.02 | 422.3 | 367.55 | 384.74 | 398.57 |
| Average number of introns per gene: | 3.65 | 2.56 | 2.32 | 4.34 | 4.29 | 4.63 | 6.26 | 6.91 | 3.95 | 3.89 | 4.15 |

**Table S5 Non-coding RNAs predicted statistics for genome assembly of ‘1380’**

| Type | Class | Number | Total length(bp) | Average length(bp) |
| --- | --- | --- | --- | --- |
| tRNA | tRNA | 573 | 41,351 | 72.17 |
| rRNA | rRNA | 2,752 | 4,501,152 | 1,635.59 |
|  | 8s | 1,592 | 183,157 | 115.05 |
|  | 18s | 570 | 1,039,711 | 1,824.05 |
|  | 28s | 590 | 3,278,284 | 5,556.41 |

**Table S6 Statistics of comparative genome analysis**

| **Type** | Number |
| --- | --- |
| Number of species | 14 |
| Number of genes | 501262 |
| Number of genes in orthogroups | 456602 |
| Number of unassigned genes | 44660 |
| Percentage of genes in orthogroups | 91.1 |
| Percentage of unassigned genes | 8.9 |
| Number of orthogroups | 36767 |
| Number of species-specific orthogroups | 14133 |
| Number of genes in species-specific orthogroups | 64625 |
| Percentage of genes in species-specific orthogroups | 12.9 |
| Mean orthogroup size | 12.4 |
| Number of orthogroups with all species present | 8236 |
| Number of single-copy orthogroups | 17 |

| Gene ID | Swissprot annotation |
| --- | --- |
| Bniv_1380G00000023840 | Caffeic acid 3-O-methyltransferase 1 OS=Ocimum basilicum OX=39350 GN=COMT1 PE=2 SV=1 |
| Bniv_1380G00000006775 | Caffeic acid 3-O-methyltransferase OS=Prunus dulcis OX=3755 GN=COMT1 PE=2 SV=1 |
| Bniv_1380G00000014290 | Caffeic acid 3-O-methyltransferase OS=Prunus dulcis OX=3755 GN=COMT1 PE=2 SV=1 |
| Bniv_1380G00000020653 | Cellulose synthase-like protein D1 OS=Arabidopsis thaliana OX=3702 GN=CSLD1 PE=2 SV=1 |
| Bniv_1380G00000006860 | Cellulose synthase-like protein D3 OS=Arabidopsis thaliana OX=3702 GN=CSLD3 PE=1 SV=1 |
| Bniv_1380G00000014590 | Cellulose synthase-like protein D3 OS=Arabidopsis thaliana OX=3702 GN=CSLD3 PE=1 SV=1 |
| Bniv_1380G00000010612 | Cellulose synthase-like protein D4 OS=Arabidopsis thaliana OX=3702 GN=CSLD4 PE=2 SV=1 |
| Bniv_1380G00000012791 | Cellulose synthase-like protein D4 OS=Arabidopsis thaliana OX=3702 GN=CSLD4 PE=2 SV=1 |
| Bniv_1380G00000013194 | Cellulose synthase-like protein D5 OS=Arabidopsis thaliana OX=3702 GN=CSLD5 PE=2 SV=1 |
| Bniv_1380G00000003598 | Laccase-4 OS=Arabidopsis thaliana OX=3702 GN=IRX12 PE=2 SV=2 |
| Bniv_1380G00000004818 | Laccase-4 OS=Arabidopsis thaliana OX=3702 GN=IRX12 PE=2 SV=2 |
| Bniv_1380G00000004982 | Laccase-4 OS=Arabidopsis thaliana OX=3702 GN=IRX12 PE=2 SV=2 |
| Bniv_1380G00000013955 | Laccase-4 OS=Arabidopsis thaliana OX=3702 GN=IRX12 PE=2 SV=2 |
| Bniv_1380G00000011942 | Protein IRX15-LIKE OS=Arabidopsis thaliana OX=3702 GN=IRX15-L PE=2 SV=1 |
| Bniv_1380G00000024859 | Protein IRX15-LIKE OS=Arabidopsis thaliana OX=3702 GN=IRX15-L PE=2 SV=1 |
| Bniv_1380G00000000407 | Transcription factor MYB52 OS=Arabidopsis thaliana OX=3702 GN=MYB52 PE=2 SV=1 |
| Bniv_1380G00000019341 | Transcription factor MYB52 OS=Arabidopsis thaliana OX=3702 GN=MYB52 PE=2 SV=1 |
| Bniv_1380G00000020914 | Transcription factor MYB52 OS=Arabidopsis thaliana OX=3702 GN=MYB52 PE=2 SV=1 |

**Table S7 Fiber growth genes in expansion genes of the *Bniv*_1380 genome**

**Table S8 Auxin signal pathway genes in expansion genes of the Bniv_1380 genome**

| Gene ID | Swissprot annotation |
| --- | --- |
| Bniv_1380G00000000885 | Auxin transporter-like protein 3 OS=Medicago truncatula OX=3880 GN=LAX3 PE=2 SV=1 |
| Bniv_1380G00000001548 | Serine/threonine-protein kinase SAPK2 OS=Oryza sativa subsp. indica OX=39946 GN=SAPK2 PE=2 SV=2 |
| Bniv_1380G00000001734 | Serine/threonine-protein kinase SAPK1 OS=Oryza sativa subsp. japonica OX=39947 GN=SAPK1 PE=1 SV=1 |
| Bniv_1380G00000002020 | Cyclin-D3-1 OS=Arabidopsis thaliana OX=3702 GN=CYCD3-1 PE=1 SV=3 |
| Bniv_1380G00000002388 | Auxin-induced protein 6B OS=Glycine max OX=3847 PE=2 SV=1 |
| Bniv_1380G00000002633 | Serine/threonine-protein kinase SAPK7 OS=Oryza sativa subsp. japonica OX=39947 GN=SAPK7 PE=2 SV=2 |
| Bniv_1380G00000003524 | Probable protein phosphatase 2C 24 OS=Arabidopsis thaliana OX=3702 GN=At2g29380 PE=1 SV=1 |
| Bniv_1380G00000004229 | Pathogenesis-related protein 1 OS=Arabidopsis thaliana OX=3702 GN=At2g14610 PE=1 SV=1 |
| Bniv_1380G00000004874 | Auxin transporter-like protein 2 OS=Medicago truncatula OX=3880 GN=LAX2 PE=2 SV=1 |
| Bniv_1380G00000004912 | Auxin transporter-like protein 2 OS=Medicago truncatula OX=3880 GN=LAX2 PE=2 SV=1 |
| Bniv_1380G00000005052 | Protein phosphatase 2C 37 OS=Arabidopsis thaliana OX=3702 GN=PP2CA PE=1 SV=1 |
| Bniv_1380G00000007347 | Indole-3-acetic acid-amido synthetase GH3.10 OS=Arabidopsis thaliana OX=3702 GN=GH3.10 PE=2 SV=1 |
| Bniv_1380G00000007760 | Shaggy-related protein kinase iota OS=Arabidopsis thaliana OX=3702 GN=ASK9 PE=1 SV=1 |
| Bniv_1380G00000009324 | Shaggy-related protein kinase eta OS=Arabidopsis thaliana OX=3702 GN=ASK7 PE=1 SV=2 |
| Bniv_1380G00000011580 | Auxin-responsive protein SAUR22 OS=Arabidopsis thaliana OX=3702 GN=SAUR22 PE=2 SV=1 |
| Bniv_1380G00000011581 | Auxin-responsive protein SAUR20 OS=Arabidopsis thaliana OX=3702 GN=SAUR20 PE=1 SV=1 |
| Bniv_1380G00000011582 | Auxin-induced protein X10A OS=Glycine max OX=3847 PE=2 SV=1 |
| Bniv_1380G00000011584 | Auxin-induced protein X10A OS=Glycine max OX=3847 PE=2 SV=1 |
| Bniv_1380G00000011585 | Indole-3-acetic acid-induced protein ARG7 OS=Vigna radiata var. radiata OX=3916 GN=ARG7 PE=2 SV=1 |
| Bniv_1380G00000011586 | Auxin-responsive protein SAUR20 OS=Arabidopsis thaliana OX=3702 GN=SAUR20 PE=1 SV=1 |
| Bniv_1380G00000011587 | Indole-3-acetic acid-induced protein ARG7 OS=Vigna radiata var. radiata OX=3916 GN=ARG7 PE=2 SV=1 |
| Bniv_1380G00000011588 | Indole-3-acetic acid-induced protein ARG7 OS=Vigna radiata var. radiata OX=3916 GN=ARG7 PE=2 SV=1 |
| Bniv_1380G00000011589 | Indole-3-acetic acid-induced protein ARG7 OS=Vigna radiata var. radiata OX=3916 GN=ARG7 PE=2 SV=1 |
| Bniv_1380G00000011590 | Auxin-induced protein X10A OS=Glycine max OX=3847 PE=2 SV=1 |
| Bniv_1380G00000011591 | Auxin-responsive protein SAUR21 OS=Arabidopsis thaliana OX=3702 GN=SAUR21 PE=2 SV=1 |
| Bniv_1380G00000011592 | Auxin-responsive protein SAUR22 OS=Arabidopsis thaliana OX=3702 GN=SAUR22 PE=2 SV=1 |
| Bniv_1380G00000011593 | Auxin-responsive protein SAUR20 OS=Arabidopsis thaliana OX=3702 GN=SAUR20 PE=1 SV=1 |
| Bniv_1380G00000011594 | Auxin-responsive protein SAUR22 OS=Arabidopsis thaliana OX=3702 GN=SAUR22 PE=2 SV=1 |
| Bniv_1380G00000011595 | Indole-3-acetic acid-induced protein ARG7 OS=Vigna radiata var. radiata OX=3916 GN=ARG7 PE=2 SV=1 |
| Bniv_1380G00000011596 | Auxin-responsive protein SAUR20 OS=Arabidopsis thaliana OX=3702 GN=SAUR20 PE=1 SV=1 |
| Bniv_1380G00000011597 | Auxin-induced protein X10A OS=Glycine max OX=3847 PE=2 SV=1 |
| Bniv_1380G00000011599 | Auxin-responsive protein SAUR20 OS=Arabidopsis thaliana OX=3702 GN=SAUR20 PE=1 SV=1 |
| Bniv_1380G00000011600 | Auxin-responsive protein SAUR20 OS=Arabidopsis thaliana OX=3702 GN=SAUR20 PE=1 SV=1 |
| Bniv_1380G00000011601 | Auxin-induced protein X10A OS=Glycine max OX=3847 PE=2 SV=1 |
| Bniv_1380G00000011602 | Auxin-induced protein X10A OS=Glycine max OX=3847 PE=2 SV=1 |
| Bniv_1380G00000011603 | Auxin-responsive protein SAUR20 OS=Arabidopsis thaliana OX=3702 GN=SAUR20 PE=1 SV=1 |
| Bniv_1380G00000011604 | Auxin-induced protein 15A OS=Glycine max OX=3847 PE=2 SV=1 |
| Bniv_1380G00000011606 | Auxin-responsive protein SAUR20 OS=Arabidopsis thaliana OX=3702 GN=SAUR20 PE=1 SV=1 |
| Bniv_1380G00000011607 | Auxin-responsive protein SAUR24 OS=Arabidopsis thaliana OX=3702 GN=SAUR24 PE=2 SV=1 |
| Bniv_1380G00000011608 | Auxin-responsive protein SAUR20 OS=Arabidopsis thaliana OX=3702 GN=SAUR20 PE=1 SV=1 |
| Bniv_1380G00000011609 | Indole-3-acetic acid-induced protein ARG7 OS=Vigna radiata var. radiata OX=3916 GN=ARG7 PE=2 SV=1 |
| Bniv_1380G00000011610 | Auxin-responsive protein SAUR20 OS=Arabidopsis thaliana OX=3702 GN=SAUR20 PE=1 SV=1 |
| Bniv_1380G00000011614 | Auxin-induced protein X10A OS=Glycine max OX=3847 PE=2 SV=1 |
| Bniv_1380G00000011616 | Indole-3-acetic acid-induced protein ARG7 OS=Vigna radiata var. radiata OX=3916 GN=ARG7 PE=2 SV=1 |
| Bniv_1380G00000011617 | Auxin-induced protein 10A5 OS=Glycine max OX=3847 PE=2 SV=1 |
| Bniv_1380G00000011618 | Indole-3-acetic acid-induced protein ARG7 OS=Vigna radiata var. radiata OX=3916 GN=ARG7 PE=2 SV=1 |
| Bniv_1380G00000011619 | Indole-3-acetic acid-induced protein ARG7 OS=Vigna radiata var. radiata OX=3916 GN=ARG7 PE=2 SV=1 |
| Bniv_1380G00000011620 | Auxin-induced protein X10A OS=Glycine max OX=3847 PE=2 SV=1 |
| Bniv_1380G00000011621 | Indole-3-acetic acid-induced protein ARG7 OS=Vigna radiata var. radiata OX=3916 GN=ARG7 PE=2 SV=1 |
| Bniv_1380G00000011622 | Auxin-induced protein X10A OS=Glycine max OX=3847 PE=2 SV=1 |
| Bniv_1380G00000011623 | Indole-3-acetic acid-induced protein ARG7 OS=Vigna radiata var. radiata OX=3916 GN=ARG7 PE=2 SV=1 |
| Bniv_1380G00000011624 | Auxin-induced protein X10A OS=Glycine max OX=3847 PE=2 SV=1 |
| Bniv_1380G00000011625 | Auxin-induced protein 15A OS=Glycine max OX=3847 PE=2 SV=1 |
| Bniv_1380G00000011626 | Indole-3-acetic acid-induced protein ARG7 OS=Vigna radiata var. radiata OX=3916 GN=ARG7 PE=2 SV=1 |
| Bniv_1380G00000011627 | Indole-3-acetic acid-induced protein ARG7 OS=Vigna radiata var. radiata OX=3916 GN=ARG7 PE=2 SV=1 |
| Bniv_1380G00000011628 | Indole-3-acetic acid-induced protein ARG7 OS=Vigna radiata var. radiata OX=3916 GN=ARG7 PE=2 SV=1 |
| Bniv_1380G00000011629 | Indole-3-acetic acid-induced protein ARG7 OS=Vigna radiata var. radiata OX=3916 GN=ARG7 PE=2 SV=1 |
| Bniv_1380G00000011630 | Auxin-responsive protein SAUR22 OS=Arabidopsis thaliana OX=3702 GN=SAUR22 PE=2 SV=1 |
| Bniv_1380G00000011631 | Auxin-responsive protein SAUR22 OS=Arabidopsis thaliana OX=3702 GN=SAUR22 PE=2 SV=1 |
| Bniv_1380G00000011632 | Auxin-induced protein 15A OS=Glycine max OX=3847 PE=2 SV=1 |
| Bniv_1380G00000011633 | Indole-3-acetic acid-induced protein ARG7 OS=Vigna radiata var. radiata OX=3916 GN=ARG7 PE=2 SV=1 |
| Bniv_1380G00000011634 | Indole-3-acetic acid-induced protein ARG7 OS=Vigna radiata var. radiata OX=3916 GN=ARG7 PE=2 SV=1 |
| Bniv_1380G00000011636 | Auxin-responsive protein SAUR22 OS=Arabidopsis thaliana OX=3702 GN=SAUR22 PE=2 SV=1 |
| Bniv_1380G00000011637 | Auxin-induced protein X10A OS=Glycine max OX=3847 PE=2 SV=1 |
| Bniv_1380G00000011640 | Auxin-induced protein 6B OS=Glycine max OX=3847 PE=2 SV=1 |
| Bniv_1380G00000011858 | Serine/threonine-protein kinase SRK2E OS=Arabidopsis thaliana OX=3702 GN=SRK2E PE=1 SV=1 |
| Bniv_1380G00000011948 | Cyclin-D3-1 OS=Arabidopsis thaliana OX=3702 GN=CYCD3-1 PE=1 SV=3 |
| Bniv_1380G00000012031 | Probable indole-3-acetic acid-amido synthetase GH3.1 OS=Arabidopsis thaliana OX=3702 GN=GH3.1 PE=2 SV=1 |
| Bniv_1380G00000013020 | Basic form of pathogenesis-related protein 1 OS=Nicotiana tabacum OX=4097 PE=3 SV=1 |
| Bniv_1380G00000013021 | Basic form of pathogenesis-related protein 1 OS=Nicotiana tabacum OX=4097 PE=3 SV=1 |
| Bniv_1380G00000013045 | Auxin-induced protein 15A OS=Glycine max OX=3847 PE=2 SV=1 |
| Bniv_1380G00000013046 | Indole-3-acetic acid-induced protein ARG7 OS=Vigna radiata var. radiata OX=3916 GN=ARG7 PE=2 SV=1 |
| Bniv_1380G00000013048 | Auxin-responsive protein SAUR27 OS=Arabidopsis thaliana OX=3702 GN=SAUR27 PE=1 SV=1 |
| Bniv_1380G00000013049 | Auxin-induced protein 10A5 OS=Glycine max OX=3847 PE=2 SV=1 |
| Bniv_1380G00000013101 | Auxin transporter-like protein 5 OS=Medicago truncatula OX=3880 GN=LAX5 PE=2 SV=1 |
| Bniv_1380G00000013118 | Pathogenesis-related leaf protein 6 OS=Solanum lycopersicum OX=4081 GN=PR1B1 PE=1 SV=2 |
| Bniv_1380G00000013455 | Auxin-responsive protein IAA20 OS=Arabidopsis thaliana OX=3702 GN=IAA20 PE=1 SV=2 |
| Bniv_1380G00000013571 | Auxin-responsive protein IAA20 OS=Arabidopsis thaliana OX=3702 GN=IAA20 PE=1 SV=2 |
| Bniv_1380G00000013932 | Auxin transporter-like protein 2 OS=Medicago truncatula OX=3880 GN=LAX2 PE=2 SV=1 |
| Bniv_1380G00000014394 | Serine/threonine-protein kinase SAPK3 OS=Oryza sativa subsp. japonica OX=39947 GN=SAPK3 PE=1 SV=1 |
| Bniv_1380G00000017993 | Basic form of pathogenesis-related protein 1 OS=Nicotiana tabacum OX=4097 PE=3 SV=1 |
| Bniv_1380G00000017994 | Pathogenesis-related protein 1 OS=Arabidopsis thaliana OX=3702 GN=PRB1 PE=2 SV=1 |
| Bniv_1380G00000021902 | Auxin-responsive protein SAUR20 OS=Arabidopsis thaliana OX=3702 GN=SAUR20 PE=1 SV=1 |
| Bniv_1380G00000021903 | Auxin-induced protein X10A OS=Glycine max OX=3847 PE=2 SV=1 |
| Bniv_1380G00000021904 | Auxin-responsive protein SAUR20 OS=Arabidopsis thaliana OX=3702 GN=SAUR20 PE=1 SV=1 |
| Bniv_1380G00000021905 | Auxin-induced protein X15 OS=Glycine max OX=3847 PE=2 SV=1 |
| Bniv_1380G00000021906 | Auxin-responsive protein SAUR20 OS=Arabidopsis thaliana OX=3702 GN=SAUR20 PE=1 SV=1 |
| Bniv_1380G00000021907 | Auxin-induced protein X15 OS=Glycine max OX=3847 PE=2 SV=1 |
| Bniv_1380G00000021908 | Auxin-responsive protein SAUR20 OS=Arabidopsis thaliana OX=3702 GN=SAUR20 PE=1 SV=1 |
| Bniv_1380G00000021909 | Auxin-induced protein 10A5 OS=Glycine max OX=3847 PE=2 SV=1 |
| Bniv_1380G00000021910 | Auxin-responsive protein SAUR20 OS=Arabidopsis thaliana OX=3702 GN=SAUR20 PE=1 SV=1 |
| Bniv_1380G00000021911 | Indole-3-acetic acid-induced protein ARG7 OS=Vigna radiata var. radiata OX=3916 GN=ARG7 PE=2 SV=1 |
| Bniv_1380G00000023879 | Probable xyloglucan endotransglucosylase/hydrolase protein 23 OS=Arabidopsis thaliana OX=3702 GN=XTH23 PE=2 SV=1 |
| Bniv_1380G00000023880 | Probable xyloglucan endotransglucosylase/hydrolase protein 23 OS=Arabidopsis thaliana OX=3702 GN=XTH23 PE=2 SV=1 |
| Bniv_1380G00000023881 | Probable xyloglucan endotransglucosylase/hydrolase protein 23 OS=Arabidopsis thaliana OX=3702 GN=XTH23 PE=2 SV=1 |
| Bniv_1380G00000023882 | Probable xyloglucan endotransglucosylase/hydrolase protein 23 OS=Arabidopsis thaliana OX=3702 GN=XTH23 PE=2 SV=1 |
| Bniv_1380G00000023883 | Xyloglucan endotransglucosylase/hydrolase protein 22 OS=Arabidopsis thaliana OX=3702 GN=XTH22 PE=1 SV=1 |
| Bniv_1380G00000023886 | Probable xyloglucan endotransglucosylase/hydrolase protein 23 OS=Arabidopsis thaliana OX=3702 GN=XTH23 PE=2 SV=1 |
| Bniv_1380G00000024538 | Serine/threonine-protein kinase SAPK2 OS=Oryza sativa subsp. indica OX=39946 GN=SAPK2 PE=2 SV=2 |
| Bniv_1380G00000024805 | Probable indole-3-acetic acid-amido synthetase GH3.1 OS=Arabidopsis thaliana OX=3702 GN=GH3.1 PE=2 SV=1 |
| Bniv_1380G00000024810 | Indole-3-acetic acid-amido synthetase GH3.3 OS=Arabidopsis thaliana OX=3702 GN=GH3.3 PE=1 SV=1 |
| Bniv_1380G00000024811 | Indole-3-acetic acid-amido synthetase GH3.4 OS=Arabidopsis thaliana OX=3702 GN=GH3.4 PE=1 SV=1 |
| Bniv_1380G00000024812 | Indole-3-acetic acid-amido synthetase GH3.3 OS=Arabidopsis thaliana OX=3702 GN=GH3.3 PE=1 SV=1 |
| Bniv_1380G00000024813 | Probable indole-3-acetic acid-amido synthetase GH3.1 OS=Arabidopsis thaliana OX=3702 GN=GH3.1 PE=2 SV=1 |
| Bniv_1380G00000024814 | Indole-3-acetic acid-amido synthetase GH3.3 OS=Arabidopsis thaliana OX=3702 GN=GH3.3 PE=1 SV=1 |
| Bniv_1380G00000024815 | Indole-3-acetic acid-amido synthetase GH3.3 OS=Arabidopsis thaliana OX=3702 GN=GH3.3 PE=1 SV=1 |
| Bniv_1380G00000024816 | Indole-3-acetic acid-amido synthetase GH3.3 OS=Arabidopsis thaliana OX=3702 GN=GH3.3 PE=1 SV=1 |
| Bniv_1380G00000024817 | Indole-3-acetic acid-amido synthetase GH3.4 OS=Arabidopsis thaliana OX=3702 GN=GH3.4 PE=1 SV=1 |
| Bniv_1380G00000024819 | Indole-3-acetic acid-amido synthetase GH3.3 OS=Arabidopsis thaliana OX=3702 GN=GH3.3 PE=1 SV=1 |
| Bniv_1380G00000024820 | Indole-3-acetic acid-amido synthetase GH3.3 OS=Arabidopsis thaliana OX=3702 GN=GH3.3 PE=1 SV=1 |
| Bniv_1380G00000024821 | Indole-3-acetic acid-amido synthetase GH3.3 OS=Arabidopsis thaliana OX=3702 GN=GH3.3 PE=1 SV=1 |
| Bniv_1380G00000024843 | Cyclin-D3-2 OS=Arabidopsis thaliana OX=3702 GN=CYCD3-2 PE=1 SV=1 |
| Bniv_1380G00000024969 | Serine/threonine-protein kinase SAPK10 OS=Oryza sativa subsp. japonica OX=39947 GN=SAPK10 PE=1 SV=1 |

**Table S9 Distribution of genes in all gene families of 12 plant species including Ramie**

| Species | Multi-copy orthologs | Other orthologs | Single-copy orthologs | Unique paralogs |
| --- | --- | --- | --- | --- |
| Arabidopsis_thaliana | 14227 | 11148 | 17 | 689 |
| Boehmeria_nivea1380 | 12361 | 9450 | 17 | 1385 |
| Boehmeria_niveaQY | 11539 | 7981 | 17 | 72 |
| Boehmeria_niveaZSZ | 11193 | 7118 | 17 | 97 |
| Camelina_sativa | 39006 | 31532 | 17 | 11439 |
| Coffea_arabica | 21820 | 15450 | 17 | 6440 |
| Fagaria_vesca | 14197 | 11208 | 17 | 5818 |
| Helianthus_annuus | 22582 | 14716 | 17 | 19810 |
| Morus_notabilis | 12843 | 7988 | 17 | 400 |
| Oryza_sativa | 14271 | 6654 | 17 | 6934 |
| Solanum_lycopersicum | 15014 | 9671 | 17 | 4644 |
| Theobroma_cacao | 12684 | 7449 | 17 | 631 |
| Vitis_vinifera | 14657 | 11734 | 17 | 4879 |
| Ziziphus_jujuba | 13697 | 9549 | 17 | 1387 |

**Table S10 The primer sequences of target and reference genes used in RT-qPCR analysis**

| **Gene ID** | Primer sequence | |
| --- | --- | --- |
|  | Forward sequence | Reverse sequence |
| *BniPAL* | TTGTGAAGCAAAACGGGCAC | TTCCGGTACTCCGCCACCAT |
| *BniHCT2* | AAAACCACCACCAAAGCAGAG | TCGGCCAGGGACTCCTTGAT |
| *Bni4CLL9* | AGCAGAAAAGAAACGCCACCA | GAGAGAAGTCGGAGTAGAGGA |
| *BniC4H2* | CCTCTTGGCGCTCTTCATTGC | CCCATGCGGAGGAGGAAGAT |
| *Bni4CL-1* | CCGCTCGAAGCAATAGGTGT | AGGCGGACCAGATCAACGTC |
| *Bni4CL-2* | CACCAAGCGAACCACGACGATCA | CTTGATCCCCAAGCGATCGAG |
| *18S* | CAGATACCGTCCTAGTCTCAACCA | CCGTGTTGAGTCAAATTAAGCCG |

**Table S11 Comparative Expression Levels of Transcription Factors in Leaves and Roots**

| ID | Leaf-1_FPKM | Leaf-2_FPKM | Leaf-3_FPKM | Root-1_FPKM | Root-2_FPKM | Root-3_FPKM | pValue | FDR | log_2_FC | regulated |  |
| --- | --- | --- | --- | --- | --- | --- | --- | --- | --- | --- | --- |
| Bniv_1380G00000025112 | 5.612109427 | 5.210204026 | 6.522130953 | 63.07482618 | 80.07269187 | 113.3518114 | 3.27E-20 | 6.17E-19 | 3.358255232 | up | bHLH |
| Bniv_1380G00000024583 | 92.66935636 | 83.38350541 | 48.96494841 | 25.98421387 | 19.9727081 | 21.00656083 | 3.86381E-08 | 1.86723E-07 | -2.395824748 | down | bHLH |
| Bniv_1380G00000020537 | 20.52594431 | 18.77650003 | 46.48948585 | 122.8798074 | 109.9187292 | 122.3748238 | 4.02375E-08 | 1.9374E-07 | 1.597849406 | up | bHLH |
| Bniv_1380G00000018062 | 14.92421249 | 17.73932252 | 23.23243187 | 78.99822854 | 74.09937225 | 83.59596586 | 1.38366E-10 | 9.13782E-10 | 1.554193235 | up | WRKY |
| Bniv_1380G00000010565 | 0 | 0.074278207 | 0 | 5.609878298 | 5.529415974 | 5.721723847 | 6.74664E-13 | 5.88241E-12 | 6.911038778 | up | WRKY |
| Bniv_1380G00000002157 | 14.63666926 | 11.88846537 | 26.22062327 | 48.20637795 | 75.54034539 | 62.44188757 | 2.67797E-05 | 8.57517E-05 | 1.346715904 | up | WRKY |
| Bniv_1380G00000024535 | 4.725553824 | 5.419224041 | 9.978359051 | 53.01942763 | 76.86955388 | 132.4351234 | 4.3571E-12 | 3.44633E-11 | 3.256791712 | up | NAC |
| Bniv_1380G00000025147 | 12.00096583 | 10.91516811 | 16.6889571 | 74.42233978 | 45.29986948 | 27.26018123 | 0.000723917 | 0.001778374 | 1.330196289 | up | NAC |
| Bniv_1380G00000024727 | 0 | 0 | 0 | 4.636524822 | 3.046682186 | 7.158128451 | 4.61691E-08 | 2.20082E-07 | 7.85523374 | up | MYB |
| Bniv_1380G00000012098 | 12.12893494 | 7.343732731 | 27.34017276 | 220.4924359 | 234.1310625 | 226.2288767 | 2.95E-20 | 5.59E-19 | 3.439649311 | up | MYB |
